# Caveolin‐1‐driven membrane remodelling regulates hnRNPK‐mediated exosomal microRNA sorting in cancer

**DOI:** 10.1002/ctm2.381

**Published:** 2021-04-08

**Authors:** Harley Robinson, Jayde E. Ruelcke, Amanda Lewis, Charles S. Bond, Archa H. Fox, Vandhana Bharti, Shivangi Wani, Nicole Cloonan, Andrew Lai, David Margolin, Li Li, Carlos Salomon, Renée S. Richards, Aine Farrell, Robert A. Gardiner, Robert G. Parton, Alexandre S. Cristino, Michelle M. Hill

**Affiliations:** ^1^ The University of Queensland Diamantina Institute The University of Queensland Woolloongabba Queensland Australia; ^2^ QIMR Berghofer Medical Research Institute Brisbane Queensland Australia; ^3^ School of Molecular Sciences The University of Western Australia Crawley WA Australia; ^4^ School of Human Sciences The University of Western Australia Crawley WA Australia; ^5^ The Harry Perkins Institute of Medical Research QEII Medical Centre Nedlands WA Australia; ^6^ University of Queensland Centre for Clinical Research, Royal Brisbane and Women's Hospital The University of Queensland Brisbane Queensland Australia; ^7^ Maternal‐Fetal Medicine, Department of Obstetrics and Gynecology Ochsner Clinic Foundation New Orleans USA; ^8^ Department of Clinical Biochemistry and Immunology, Faculty of Pharmacy University of Concepción Concepción Chile; ^9^ Institute for Molecular Bioscience The University of Queensland St Lucia Queensland Australia; ^10^ Griffith Institute for Drug Discovery Griffith University Brisbane Queensland Australia; ^11^ Centre for Microscopy and Microanalysis The University of Queensland St Lucia Queensland Australia

**Keywords:** cancer, cargo, epigenetics, extracellular vesicle, hnRNPK, lipid raft, membrane raft, miRNA, RNA‐binding protein, RNA sequence motif

## Abstract

**Background:**

Caveolae proteins play diverse roles in cancer development and progression. In prostate cancer, non‐caveolar caveolin‐1 (CAV1) promotes metastasis, while CAVIN1 attenuates CAV1‐induced metastasis. Here, we unveil a novel mechanism linking CAV1 to selective loading of exosomes with metastasis‐promoting microRNAs.

**Results:**

We identify hnRNPK as a CAV1‐regulated microRNA binding protein. In the absence of CAVIN1, non‐caveolar CAV1 drives localisation of hnRPNK to multi‐vesicular bodies (MVBs), recruiting AsUGnA motif‐containing miRNAs and causing their release within exosomes. This process is dependent on the lipid environment of membranes as shown by cholesterol depletion using methyl‐β‐cyclodextrin or by treatment with n‐3 polyunsaturated fatty acids. Consistent with a role in bone metastasis, knockdown of hnRNPK in prostate cancer PC3 cells abolished the ability of PC3 extracellular vesicles (EV) to induce osteoclastogenesis, and biofluid EV hnRNPK is elevated in metastatic prostate and colorectal cancer.

**Conclusions:**

Taken together, these results support a novel pan‐cancer mechanism for CAV1‐driven exosomal release of hnRNPK and associated miRNA in metastasis, which is modulated by the membrane lipid environment.

## Graphical abstract

Highlights

1. Non‐caveolar caveolin‐1 drives non‐canonical localization of hnRNPK to multivesicular bodies (MVB) via membrane raft‐dependent mechanism.

2. MVB localized hnRNPK mediates EV loading of AsUGnA motif‐containing miRNA, which is required for EV‐induced in vitro osteoclastogenesiss.

3. Elevated EV hnRNPK in biofluids from metastatic cancer patients suggest a role as a biomarker.

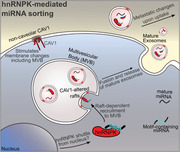

## 1 INTRODUCTION

Caveolae are a flask‐shaped subtype of membrane raft formed by the interaction of structural membrane protein caveolin‐1 (CAV1) and cytoplasmic protein cavin‐1 (CAVIN1) (also called PTRF, polymerase I and transcript release factor) in a cholesterol‐ and sphingolipid‐rich membrane.^1^ While these proteins are co‐expressed in healthy human cells, advanced prostate cancer cells express CAV1 without CAVIN1, leading to aberrant pro‐metastatic non‐caveolar CAV1 domains.^2^ We have utilised prostate cancer cell line models to assess the roles of these caveolae proteins in regulating EV cargo and disease pathology.

The expression pattern CAV1+/CAVIN1‐ is replicated in the non‐caveolar PC3 (aggressive prostate cancer) cell line. Ectopic expression of CAVIN1 in PC3 cells (CAV1+/CAVIN1+ expression, named here PC3‐CAVIN1) not only rescues caveolae formation, but attenuates multiple oncogenic properties in vitro and in vivo.^2^, ^3^ In addition to suppressed growth and migration, PC3‐CAVIN1 cells display an altered tumour microenvironment in a xenograft model, including reduced stromal myofibroblasts and attenuated metastasis to lung and bone.^4^ Mechanistically, this was found to be due to altered extracellular vesicle (EV) content affecting osteoblast proliferation and differentiation.^4^

EVs are membrane‐enclosed particles that are naturally released from a cell. They are reported to transfer functional messenger RNAs (mRNAs), microRNA (miRNAs) and proteins to recipient cells and thereby change their cellular processes.^5^ Two major types of EVs have been defined based on the route of release: exosomes are generated in the endocytic system in multi‐vesicular bodies (MVB) and are released via fusion of the delimiting membranes of the MVB with the plasma membrane, while larger microvesicles pinch off the cell surface.^5^ In this paper, we follow the recommendation of the International Society of Extracellular Vesicles, we have used the term 'exosome' when we know the mode of biogenesis, and the term 'EV' to denote a mixture of vesicles or where mode of biogenesis is not clear.^6^ Regardless of the mode of biogenesis, both vesicle‐based communication systems appear to be exquisitely regulated in normal physiology but are altered in diseases, including cancers.^7^ Ultimately, the pathological or physiological effects delivered by EVs are largely dependent on the cargo.

In recent years, the RNA content of EVs, particularly the microRNA (miRNA) subpopulation, has been increasingly implicated in disease pathology.^8^ miRNAs are key regulatory molecules that control the expression of several genes by directly binding to mRNA to suppress protein translation,^9^ which ultimately results in altered pathway activity. While studying PC3 EV miRNA as a modulator of osteogenic activity, we made the intriguing observation that the osteogenic miR‐148a‐3p was reduced in CAVIN1 PC3 EVs without altering total cellular levels.^4^ Although CAVIN1 expression alters the PC3 membrane raft and EV proteome,^3^ CAVIN1 itself was not detected in EV,^4^ suggesting that CAVIN1 indirectly regulates EV miRNA content.

EV cargo can be ascertained passively from the cytoplasm during biogenesis, or via regulated sorting mechanisms. Protein cargos are loaded to EVs via endosomal sorting complexes required for transport complex and tetraspanin‐based mechanisms.^10^ EV membranes are enriched in cholesterol and sphingolipids,^11^ a property shared with caveolae membranes and membrane raft fractions that are obtained as detergent‐resistant membranes (DRMs). In contrast to the surrounding membrane, membrane rafts possess differing biophysical properties, such as increased bilayer thickness and viscosity, which drive lateral lipid and protein compartmentalisation.^12^ EV membrane lipid composition has been implicated in protein^13^, ^14^ and RNA cargo sorting,^15^ although the mechanisms are yet to be elucidated.

As caveolae formation impacts membrane raft proteome,^1^ we hypothesised that CAVIN1 regulates miRNA loading to EVs by modulating RNA‐binding proteins in the membrane raft. To test this hypothesis, we sought to identify RNA‐binding proteins responsible for selective EV miRNA loading and target miRNA sequence motif/s, using an integrated approach combining small RNA sequencing, computational and cell‐based analyses. We identified heterogeneous nuclear ribonucleoprotein K (hnRNPK) as the central mediator of CAV1‐driven sequence‐specific miRNA sorting. While hnRNPK is a multi‐functional nuclear protein with known healthy and disease functions, this study provides the first documented role of exosomal hnRNPK and its direct interaction with miRNA in cancer. Furthermore, regulation of miRNA sorting via membrane remodelling driven by non‐caveolar CAV1 provides integrated mechanistic understanding of exosomal cargo loading.

## 2 MATERIALS AND METHODS

### 2.1 Human subjects and samples

For the prostate cancer cohort, ethical approval was obtained from the University of Queensland Medical Research Ethics Committee (project number: 2004000047) and the Royal Brisbane and Women's Hospital Human Research Ethics Committee (HREC/09/QRBW/320, HREC/09/QRBW/305 together with 1995/088B). Informed consent was obtained from all participants. Initial and updated biopsy and radical prostatectomy (RP) histology specimens were reviewed by expert uropathologists. Risk stratification for biopsies was performed using the D'Amico criteria recommended in the American Urological Association Guidelines. Seminal fluid specimens collected into sterile urine jars containing 20 ml Hanks Balanced Salt Solution were layered over a 10 ml isotonic Percoll gradient (GE Healthcare‐Pharmacia) and centrifuged at 974 × g for 30–60 min at 4°C. The non‐sperm epithelial cell layer was harvested for cell‐based analyses as previously reported, while supernatants (seminal plasma) were collected in 1 ml aliquots, snap‐frozen on dry ice and stored at −80°C. All samples used for this study were assessed as clinically significant at follow‐up. Progressively rising serum prostate‐specific antigen levels post‐therapy (RP, androgen‐deprivation therapy, external beam therapy) indicated the presence of non‐localised, metastatic disease at the outset.

The colorectal cancer study was approved by Ochsner Clinic Foundation investigative review board and conducted in accordance with the ethical standards of the Institutional Committee on Human Experimentation. Patients undergoing resection for colorectal adenocarcinoma were invited to participate. Tissue and blood samples were collected from consenting patients at surgery. A board‐certified pathologist determined the final diagnosis, tumor type and grade. Plasma samples were isolated from whole blood and stored at −20°C prior to experiments.

Both patient cohorts information can be found in Table S1 (prostate cancer cohort, Tab 1; colorectal cancer cohort, Tab 2).

### 2.2 Cell culture

#### 2.2.1 Source and general culture conditions

HEK293 cells were a generous gift from the Simpson laboratory, University of Queensland Diamantina Institute (UQDI), Faculty of Medicine, The University of Queensland. PC3 cell lines were cultured in 5% FBS/RPMI1640 media (Bovogen, Gibco) at 37°C in a 5% CO_2_ incubator (generation and characterisation described by Moon et al^2^). LNCaP cell lines (generation and characterisation described in^2^) were cultured in 8% FBS/RPMI1640 media (Bovogen, Gibco), and HEK293 cell lines were cultured in 10% FBS/DMEM media (Bovogen, Gibco) in the same conditions. Stable expression of GFP (‐CONT cells) and GFP tagged CAVIN1 (‐CAVIN1 cells) or CAV1 (‐CAV1 cells) in cell lines were maintained in 0.1 mg/ml G418 (Gibco). RAW264.7 cells were a generous gift from the Anderson laboratory (Iron Metabolism, QIMRB) and grown in 10% FBS/DMEM.

#### 2.2.2 Generation of CAV1‐HEK293 cells

Lentiviral particles for GFP tagged CAV1, GFP and control (pLV411) were obtained from UQDI ARVEC facility, and cells were transduced as previously described.^16^ Transduced cells were selected by GFP expression using fluorescence‐activated cell sorting.

#### 2.2.3 Transient knockdown using siRNA

Wild type PC3 cells were grown in 10 cm dishes until 80% confluency prior to siRNA treatment, using three dishes per condition, per replicate. Transfection of hnRNPK or non‐targeting ON‐TARGETplus SMARTpool siRNA (Dharmacon) was completed as per manufacturer's instruction. Briefly, lipofectamine 2000 was used to transfect 100 nM siRNA into PC3 cells in serum‐free conditions. After 24 h incubation, conditioned media was collected for EV extraction.

#### 2.2.4 Cell line treatments

Cells were grown in 10 cm plates (for subcellular fractionation) or in six‐well plates with coverslips (for microscopy) until 70% confluency in normal growth conditions. Media was removed, and cells were washed with PBS, followed by addition of membrane raft disrupting reagent in RPMI‐1640 media containing 5% lipoprotein deficient serum (LPDS). After 24 h incubation, cells were fixed in 4% paraformaldehyde (PFA) for microscopy, or harvested using a cell scraper then transferred to Eppendorf tubes. Cell pellets were stored at −20°C.

#### 2.2.5 Osteoclastogenesis assay

One thousand RAW264.7 were seeded into 24 well plates in normal growth conditions. After 24 h, 10 μg/mL EVs from hnRNPK‐knockdown or non‐targeting siRNA treated PC3 cells, or EV‐depleted media (50μL per well), or positive control (40 ng/mL RANKL), or PBS (50μL) negative control were added. After 72 h, media containing treatments were refreshed. After a further 48 h, cells were fixed in 4% PFA and TRAP stained for 1 h at 37°C using 2.5 mM Naphthol‐ASBI phosphate, 0.5 mM Fast Red Violet LB, 0.1 M sodium acetate, 50 mM sodium tartrate and 1% Triton‐X100. Using light microscopy (40x magnification), 10 fields were captured for each well and multinucleated TRAP‐positive were counted. Three biological replicates were completed using EVs collected from three separate knockdown experiments.

### 2.3 EV purification

#### 2.3.1 EV purification from culture supernatant

EVs were isolated from cultured cells using ultrafiltration and ultracentrifugation. Briefly, cells were seeded and grown in appropriate growth media in 15‐cm dishes until 70% confluency. Growth media was removed, and cells were gently washed three times with PBS, to remove FBS EVs, before serum free RPMI1640 (Gibco) was added. After incubation for 24 h, the supernatant was collected, and whole cells and debris were removed by centrifugation at 800 x g for 5 min at 4°C and subsequently 5,000 x g for 10 min at 4°C. Cleared supernatant was then concentrated using Amicon 10 kDa spin column units (Millipore) until 1 ml of concentrated media remained. Concentrated media was transferred to ultracentrifuge microfuge tubes, and EVs were extracted at 100,000 x g, 2 h, 4°C. EV pellets were washed with cold PBS, resuspended in PBS and stored at −80°C. EVs isolated from PC3 cell lines were previously characterised.^2^

#### 2.3.2 EV purification from plasma and seminal plasma

Plasma was diluted with an equal volume of PBS and centrifuged at 2,000 x g for 30 min at 4°C to pellet any cellular debris. The supernatant was centrifuged at 12,000 x g for 45 min at 4°C to pellet larger vesicles. The supernatant was transferred to an ultracentrifuge tube and centrifuged at 100,000 x g for 2 h to pellet EVs. The 100,000 x g pellet was resuspended in PBS and filtered through a 0.22 μm filter to break up any clumps and centrifuged again at 100,000 x g for 2 h. The EV pellet containing the enriched exosome population is resuspended in PBS and at −80°C until later use.

To collect EVs from seminal plasma, a 1 ml aliquot of dilute seminal fluid supernatant was centrifuged at 100,000 x g for 60 min, and resulting pellets were washed using PBS and stored at −20°C until use.

### 2.4 Small RNA‐sequencing and differential abundance analysis

Total RNA from cells and EVs were isolated by Trizol (Invitrogen) extraction, carried out at room temperature (approximately 22°C) as per manufacturer's instruction. Resuspended RNA was quantified using a Nanodrop Spectrophotometer to ensure equal quantities of RNA for RNA‐seq analysis.

Small RNAs were selected for sequencing using the NEBNext Small RNA library Prep Kit. Libraries were size selected using a Perkin Elmer Labchip XT. Whole cell and EV libraries were multiplexed from three biological replicates and sequenced on an Illumina NextSeq mid‐output run at a read depth of one million reads per sample. Reads were mapped to miRBase (v21), resulting in raw count data. The RNA sequencing data have been deposited to the Gene Expression Omnibus under the accession number GSE109356.

miRNA counts were filtered to remove low abundant miRNA with less than 20 counts identified in total across all replicates, and remaining data were normalised using the empirical normalisation method from the RUVseq package in R studio.^17^ This normalisation method assumes RNA amount is equal across the samples as an alternative to RNA‐spike in normalisation methods (no spike in used here). Data sets were split into EV (resulting in log_2_FC EV) and cellular data (log_2_FC Cell) for subsequent processing. The normalised counts were processed using DESeq2 R software package using the standard pipeline to compare the three biological replicates for each condition.^18^ miRNAs consistently identified in EVs were compared to their cellular levels to determine selective export by calculating fold enrichment (FE) where FE = log_2_FC EV – log_2_FC Cell. Graphpad Prism 7 was used to generate heat maps and scatterplots.

### 2.5 RNA motif discovery and enrichment analysis

Mature miRNA sequences were acquired from miRBase (v21). MEME (Multiple EM for Motif Elicitation) program was used for the de novo discovery of RNA motifs significantly overrepresented in selectively exported miRNA sequences with parameters adjusted to zero or one motif occurrence per sequence (‐mod zoops), minimal motif length equals 4 (‐minw 4), maximal motif length equals 10 (‐maxw 10) and 0‐order background frequencies of nucleotides based on input miRNA sequences. Small motifs were predicted (4–10 nucleotides) due to the small size of mature miRNAs. Motifs identified are returned with an E‐value to describe the likelihood of a random occurrence of this motif in the inputted sequences, where E‐values < 0.05 are considered statistically significant, and not occurring by chance. Enrichment analysis of motifs discovered in the selectively exported miRNA sequences (all EV miRNAs used as background) was performed using the algorithms implemented in TAMO package.^19^ Church values and *p*‐values were calculated in the TAMO package, as two different representations of statistical significance of the motifs.^20^ Position‐specific scoring matrices of other RNA‐binding proteins were acquired from RNA‐Binding Protein database.^21^ Transcript targets for the motif containing microRNAs were identified using miRTarBase and applied to PANTHER and Reactome online software.^22^, ^23^

### 2.6 Immunoprecipitation

Cells were lysed in cold hypotonic lysis buffer (10 mM Tris‐Cl [pH 7.5], 20 mM KCl, 1.5 mM MgCl_2_, 5 mM DTT, 0.5 mM EDTA, 5% glycerol, 0.5% NP‐40) supplemented with 40 U/μL RNase OUT (ThermoFisher) and protease inhibitors (Aprotinin, Antipain, Pepstain A, Leupetin, Benzamidine) for RNA‐immunoprecipitation. Resulting lysate was incubated at 4°C for 1 h with 1 nmol 3′ biotinylated miRNA oligonucleotide with gentle rotation to avoid settling. Reaction mixture was added to washed M‐270 streptavidin Dynabeads and incubated for a further 90 min at 4°C. Supernatant was subsequently removed and used to confirm target presence in reaction. Beads were washed five times with lysis buffer before incubation at 95°C for 5 min in SDS‐PAGE buffer to elute protein for subsequent western blotting. Quantities were determined using the LI‐COR Odyssey software to measure hnRNPK band intensity and normalised to total intensity across the replicate to account for differences in blotting efficiency. The target oligonucleotide was designed to mimic mature miR‐148a‐3p sequence, with a 3′ biotin modification. The negative control oligonucleotide was generated using GenScript scramble sequence generator and compared to known miRNA sequences using miRBase's search by sequence function to confirm no possible mimic (all oligonucleotide sequences available in Table S6).

Co‐immunoprecipitation experiments to determine hnRNPK binding partners were performed using Pierce magnetic Protein A/G beads, as per manufacturer's instruction. Eluent was collected after boiling with SDS‐PAGE buffer.

### 2.7 Western immunoblotting

Proteins were separated on 12.5% acrylamide/bisacrylamide gels with 4% stacking gel and transferred to Immobilon‐P PVDF membranes (Merck Millipore) for 1 h at 100 V. Membranes were blocked using 3% BSA/TBS buffer and incubated with diluted primary antibodies (1 μg/ml) and LI‐COR IRdye secondary antibodies (0.75 μg/ml).

### 2.8 In vitro microscale thermophoresis assay

To produce recombinant hnRNPK for in vitro microscale thermophoresis (MST) assay, a vector encoding the Homo sapiens hnRNPK gene was obtained and sub‐cloned into a pETM‐11 backbone engineered with an N‐terminal 6xHis‐GFP tag and tobacco etch virus recognition sequence as per.^24^ Dilution series of recombinant hnRNPK or negative binding control BSA (NanoTemper Technologies) starting at 150 μM were prepared in 50 mM HEPES, 0.3 M KCl, pH 7.4 and mixed with 5′ 6‐FAM labelled RNA oligonucleotides (integrated DNA technologies) to a final RNA concentration of 40 nM. The temperature jump data from three replicate measurements were averaged and analysed using the implemented fitting software NT Analysis (NanoTemper Technologies). Binding isotherms were fitted using the Hill equation. MST measurements were performed in standard treated capillaries (NanoTemper Technologies) on a Monolith NT.115 system (NanoTemper Technologies) using 40% LED and 40% IR‐laser power. Laser on and off times were set at 30 s and 5 s, respectively. RNA probe names and sequences used in these experiments were designed to mimic wild type miR‐148a‐3p sequence, or mimic with transition mutations in the motif region (all oligonucleotide sequences are available in Table S6).

### 2.9 Microscopy

#### 2.9.1 Immunofluorescence microscopy

Cells were grown to 70% confluency on coverslips prior to fixation with 4% PFA for 30 min at room temperature. All further incubations were performed at room temperature. After washing with PBS, 0.1% Triton‐X100 in blocking solution (1% BSA/PBS) was added to the coverslips to block and permeabilize the cells. Cells were incubated with desired primary antibodies for 1 h followed by washing three times with PBS and incubating with secondary AlexaFluor antibodies for an additional 1 h. Nuclei were stained using 1:1000 dilutions of DAPI (4′,6′‐diamidino‐2‐phenylindole, Sigma) and mounted using Prolong Diamond mounting media (Invitrogen).

#### 2.9.2 miRNA in situ hybridisation

Cells were grown to 70% confluency on coverslips and were fixed using 100% cold methanol in preparation for miRNA in situ hybridisation (as described in Robinson et al^25^). Negative control, Cy5‐scrambled‐miR‐148a oligo, was generated using the GenScript scramble sequence generator and compared to known miRNA sequences using miRBase's search by sequence function to confirm no known predicted target (all oligonucleotide sequences are available in Table S6).

#### 2.9.3 Co‐localisation analyses

Two different co‐localisation experiments were conducted using the same statistical method and processing. Images obtained from the immunofluorescence and miR‐ISH experiments were analysed with the Coloc2 analysis on the ImageJ (FIJI) software. Given that hnRNPK is known to be present in multiple localisations (nucleus and cytoplasmic regions), we used region of interest (ROI) analysis to focus on the areas where the miRNA probe or the MVB marker was in high concentration. A total of 10 ROI were used for each condition across at least three biological replicates, using two–three cells per replicate.

### 2.10 Subcellular fractionation

Cell pellets for cytoplasm/nucleus fractionation were resuspended in a hypotonic extraction buffer (10 mM Tris pH 7.5, 10 mM NaCl, 0.5 mM ETDA, and protease inhibitor cocktail) and incubated on ice for 30 min. Crude extraction of nuclear and cytoplasmic extraction was completed by passing the sample 20 times, although a 24 g needle, and nuclear fraction extracted by centrifugation at 1000 x g for 30 min. Supernatant was collected as cytoplasmic fraction, and pelleted nuclear fraction was washed with PBS.

Cell pellets for detergent resistant membrane (DRM) extraction were extracted as per Inder et al.^3^ Five fractions were collected; sucrose percentage (refractometer) and protein concentration (BCA assay, Pierce) were recorded.

### 2.11 ddPCR of EV miRNA

RNA was extracted from EV pellets using mirVana miRNA isolation with enrichment for small RNA. Equal amount of RNA was processed for reverse transcription, using miRcury LNA RT kit. Samples were then diluted 1:50 and prepared for ddPCR using EVA green SYBR mix and commercial LNA miRNA probes, as per manufacturer's instruction. The PCR mix was partitioned into droplets before conducting PCR, and ddPCR results acquired using the QX200 Digital Droplet system (BioRad) and recommended protocol. Quantasoft software determined concentration of each target (Poisson corrected copies/μL).

### 2.12 Proteomics

Sequential Windowed Acquisition of All Theoretical Fragment Ion Mass Spectra (SWATH) proteomics was conducted for colorectal cancer patient plasma EV. For comprehensive information related to SWATH analysis, see supporting methods and materials.

Briefly, EV samples were prepared using a standard trypsin (Promega) digest method, using dithiothreitol (Sigma) and iodoacetamide (Sigma). Note that 2 μg peptide was analysed in data‐dependent acquisition mode using a SCIEX Triple TOF spectrometer (ABSCIEX, Redwood City, CA, USA) coupled to a Nano Ultra 1D+ HPLC system (Eksigent, Redwood City, CA, USA). For SWATH processing, the SWATH Acquisition Microapp (version 2.0) within PeakView (RRID: SCR_015786; version 2.2) was used to align samples with the SWATH library.

A total of 16 colorectal cancer plasma EV were analysed, four patient samples was selected for each grade I to IV, then grouped as early (grades I and II) or metastatic (grades III and IV), making *n* = 8 per group. Fold changes were generated using the mean normalised intensity for each protein comparing between early and metastatic colorectal cancer (CRC), and log_2_ transformed. For volcano plot, p‐values were converted to ‐log_2_p‐values and plotted against log_2_FC using R studio. Statistically significant proteins (*p*‐value < 0.05) were coloured red, and those proteins increased in metastatic CRC (positive log2FC value) were labelled with gene name for visibility. Using GraphPad Prism, early and metastatic CRC‐normalised intensities for hnRNPK were graphed using box and whisker plots which displays range of observation, median and upper and lower quartiles.

### 2.13 Statistical analysis

Data are presented as means ± SEM unless otherwise stated in figure legends. Statistical analysis using two‐sided Mann‐Whitney test was completed using GraphPad Prism (v7) or Excel, where applicable. Correlation analysis was completed using ImageJ Coloc2 function (Pearson correlation, no threshold). RNA‐seq data were analysed using DESeq2. This package as default considered *p*‐values < 0.05 statistically significant for *t* test and false discovery rate corrected data. Default statistical tests, including two‐sided *t* test and Church scores, were acquired from TAMO and MEME suite packages, where applicable. Exact *p*‐values, *n* values and statistical test used are shown in figures and figure legends.

## 3 RESULTS

To test the hypothesis that CAVIN1 regulates miRNA loading to EVs by modulating RNA‐binding proteins in the membrane raft proteome, we began by identifying a repertoire of selectively loaded miRNAs and a candidate miRNA binding protein in the EV and raft proteome regulated by CAVIN1 expression.

### 3.1 CAVIN1 expression in PC3 cells selectively regulates EV miRNA loading

**FIGURE 1:**
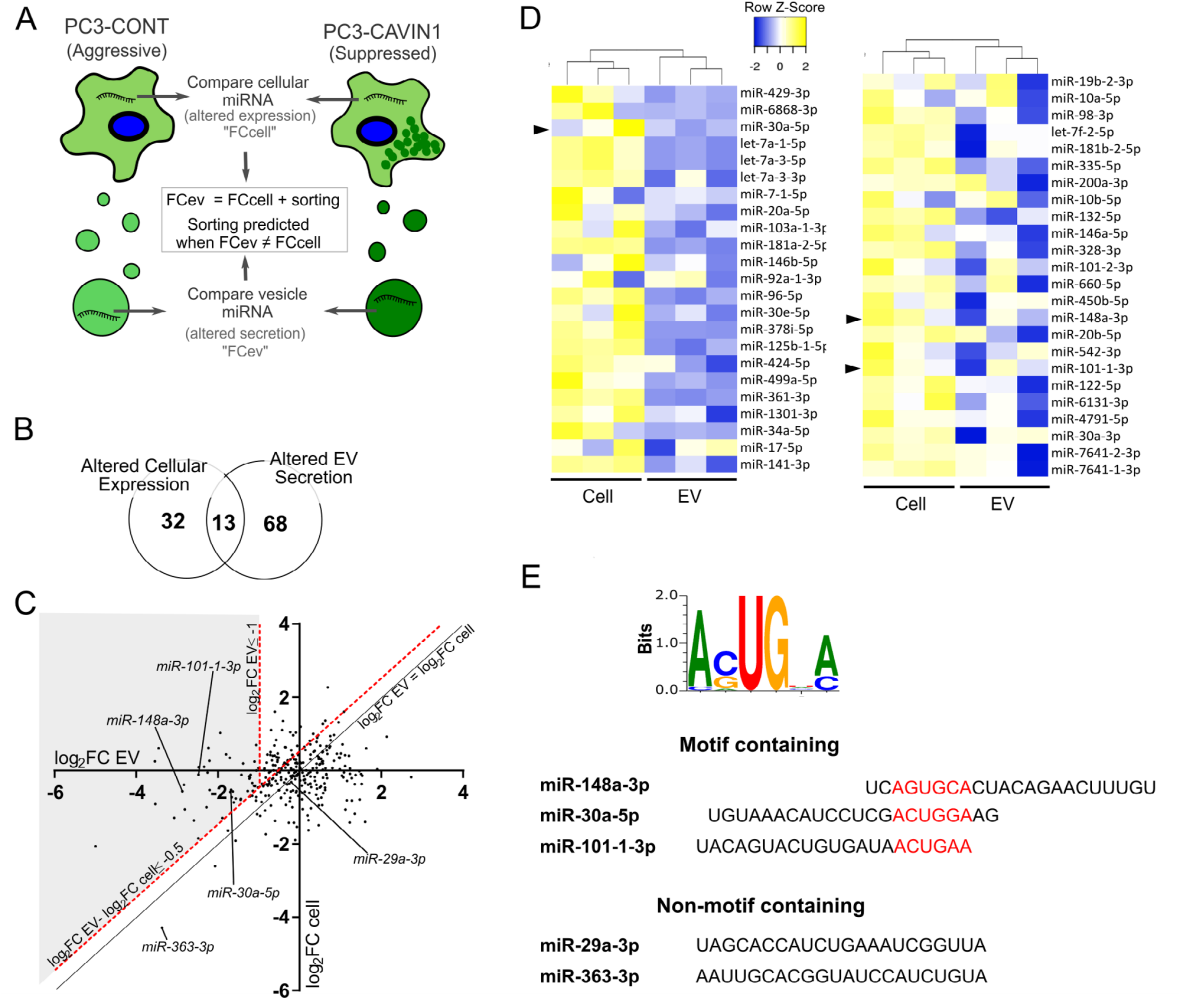
CAVIN1 selectively regulates EV miRNA content in PC3 cells. (**A)** Experimental design aimed to identify miRNAs selectively exported to EVs using small RNA sequencing data. DESeq2 analysis was conducted on miRNA counts for EV and cell content, where log_2_ fold change (log_2_FC) was calculated between PC3‐CONT and PC3‐CAVIN1 cells and their respective EVs (*n* = 3). (**B**) Comparison of miRNAs altered in cells and EVs. Data shown are number of cellular (expression changes) and EV miRNAs (secretion changes) with a log_2_FC > 1, and ← 1. (**C)** Scatter plot of log_2_FC in EV and cell for each miRNA (represented by a dot). Diagonal black line represents equivalent change in EV and cellular miRNA levels upon CAVIN1 expression. Red dotted lines indicate log_2_FC EV of −1 and log_2_FC EV less than log_2_FC cell by −0.5. Selected miRNAs are indicated by shaded area. (**D)** Heatmap showing row normalised log_2_FC of the 47 selectively exported miRNAs. Black arrows mark miRNA of focus in other assays. (**E)** Sequence motif enriched in the selective exported miRNA data set, with positional alignment of the identified motif in motif‐containing miRNA used for subsequent experiments

Previously we used a candidate approach to demonstrate that CAVIN‐1 induced selective reduction of miR‐148a‐3p in PC3 EV but not in the cell, while miR‐125a‐3p was not significantly altered in either compartment.^4^ To determine the broader applicability of the observation, we used RNA sequencing to map the repertoire of selectively exported miRNA associated with CAVIN1 expression in PC3 cells. As depicted in Figure 1A, EV miRNAs with their respective cellular miRNA content from PC3‐CAVIN1 cells (CAV1+/CAVIN1+) and control PC3‐CONT cells (CAV1+/CAVIN1‐) were analysed. A total of 317 miRNAs were identified in both cells and EVs over three biological replicates. To evaluate the CAVIN1‐induced changes in EV and cellular miRNA levels, log_2_ fold change (log_2_FC) values were generated for EV and cell fractions (Table S2, Tabs 1 and 2). Overlap analysis showed that a large proportion of miRNAs with altered EV abundance (fold change increased or decreased by two‐fold) tends not to be similarly altered in cellular expression (Figure 1B), supporting the presence of sorting mechanisms regulating the EV miRNA content independent of cellular expression changes.

To define the repertoire of selectively loaded miRNA in this system, we plotted CAVIN1‐dependent log_2_FC_EV_ compared to log_2_FC_cell_, where passively sampled miRNAs are expected to exhibit equivalent log_2_FC in EVs and cells (black line in scatterplot; Figure 1C), while selectively loaded miRNAs will show CAVIN1‐dependent change (increase or decrease) in the log_2_FC_EV_ compared to log_2_FC_cell_. Based on a combined threshold of at least a twofold decrease in EV from PC3‐CAVIN1 cells compared to PC3‐CONT (log_2_FC_EV_ ≤‐1) and at least a two‐fold depletion in EV compared to cell (log_2_FC_EV_ – log_2_FC_cell_ ≤ ‐0.5, indicated by shaded area in Figure 1C), a total of 47 miRNAs were deemed to be selectively down‐regulated by CAVIN1 via a selective export mechanism (Figure 1D, Table S2, Tab 3). As expected, the list includes miR‐148a‐3p, previously shown to be selectively down‐exported in PC3‐CAVIN1 EVs by quantitative reverse transcription polymerase chain reaction (qRT‐PCR).^4^

Next, we used the mature sequences of the 47 selectively exported miRNAs for de novo RNA motif discovery using expectation‐maximisation algorithms.^26^ A single RNA motif, AsUGnA, was found (Figure 1E, E‐value = 0.019), which is significantly enriched in CAVIN1‐inhibited selectively exported miRNAs compared to all 317 cellular miRNAs (enrichment score = 4.12; *p*‐value = 7.5e‐05, Table S2, Tab 4). For comparison, enrichment of the binding motifs previously identified for miRNA sorting proteins, hnRNPQ and hnRNPA2B1, were also determined and showed no enrichment in our selectively exported miRNAs (Table S2, Tab 4). Of the 47 selectively exported miRNAs down‐regulated by CAVIN1, 21 miRNAs contain sequences with over 70% similarity to the identified AsUGnA motif (Table S2, Tab 5), with the known CAVIN1 down‐regulated miR‐148a‐3p showing 85% similarity to the identified AsUGnA motif (Table S2, Tab 5). Alignment of these sequences shows no positional preference for the motif along the mature sequence (Table S2, Tab 5, Columns C and D).

We then conducted pathway enrichment analysis on the targets of the 21 AsUGnA motif‐containing miRNAs that were down‐regulated by CAVIN1 in PC3‐CAVIN1 (CAV1+/CAVIN1+) EVs. This revealed the protein targets mediated by these miRNA are enriched for immune responses and angiogenesis GO terms, as well as pathways related to proliferation and adhesion, such as TGF, EGF, FGF, MAPK and NOTCH pathways (Table S2, Tab 6). This suggested that motif‐directed miRNA loading into EVs may promote pro‐metastatic pathways in target cells.

### 3.2 hnRNPK subcellular localisation is regulated by CAV1 and CAVIN1

**FIGURE 2:**
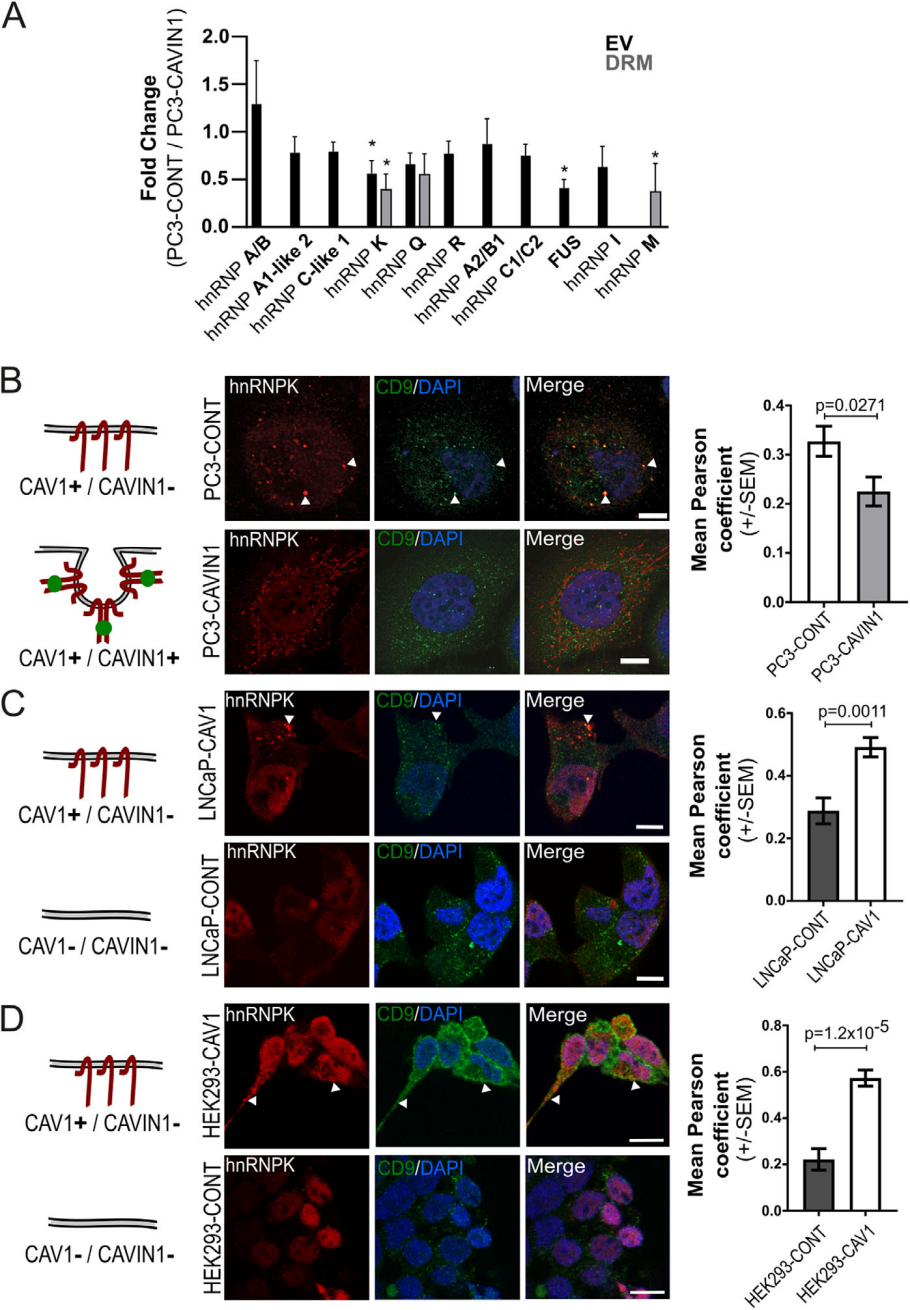
Non‐caveolar CAV1 regulates hnRNPK localisation. **(A)** hnRNP family member abundance in EV and DRM fractions shown as fold change between PC3‐CAVIN1 and PC3‐CONT cells (CAVIN1/CONT) originally reported in.^3^ *n* = 3, error bars represent standard deviation. *p ≤ 0.05. (**B–D)** Diagrams (*left*) depict the CAV1 and CAVIN1 status of each cell line used. Confocal immunofluorescent microscopy of hnRNPK (red) and CD9 (green) MVB marker in three cell systems: (**B**) PC3 cell line with and without CAVIN1 (PC3‐CAVIN1 and PC3‐CONT, respectively); ectopically expressed CAV1 in LNCaP (**C**) and HEK293 (**D**) cell lines. White arrows point to example areas of co‐localisation. All scale bars indicate 10 μm. Quantitative co‐localisation analysis *(right)* was performed using Pearson correlation on 10 regions of interest for each condition from three to four biological replicates (composite images for all replicates used in co‐localisation analysis available in Figure S2, S3 and S4). A two‐sided unpaired Mann‐Whitney test was used to compare Pearson coefficient values between the conditions

To identify RNA binding proteins down‐regulated by CAVIN1 in both EV and membrane raft fractions, we made use of our published PC3/PC3‐CAVIN1 quantitative subcellular proteomics dataset.^3^ Searching for RNA‐binding proteins (GO: 0003723) that were down‐regulated in both EV and DRM identified a single protein: heterogeneous nuclear ribonucleoprotein K (hnRNPK). This was intriguing, as two other members of the hnRNP family, hnRNPA2B1 and hnRNPQ, were recently reported to mediate miRNA loading to EVs.^27^, ^28^ This prompted us to inspect the proteomics data for all members of the hnRNP family, revealing that only hnRNPK was significantly altered in both fractions by CAVIN1 expression (Figure 2A).

hnRNPK is predominantly located in the nucleus in normal cells but has been reported to translocate to the cytoplasm in some cancer cells.^29^, ^30^ In prostate cancer, hnRNPK localisation was previously reported to regulate androgen receptor (AR) activity and prostate cancer prognosis.^31^, ^32^, ^33^ Nuclear hnRNPK was reported to be part of a transcriptional repressor for AR gene^31^ and correlated with Gleason grade and poor prognosis.^32^ On the other hand, cytoplasmic hnRNPK was proposed to down‐regulate AR mRNA translation in prostate cancer.^33^ The detection of hnRNPK in PC3 EV led us to hypothesize that hnRNPK translocates to MVB together with miRNA cargo, becomes incorporated into intraluminal vesicles and secreted in exosomes. To begin assessing this, we performed immunofluorescence co‐localisation of hnRNPK with the MVB marker CD9.

In PC3‐CONT cells, hnRNPK was detected in cytoplasmic puncta that co‐localised with CD9 (Figure 2B). Co‐localisation with CD9 was lost in PC3‐CAVIN1 cells (Figure 2B), with hnRNPK instead co‐localising with endoplasmic reticulum and mitochondria (Figure S1, Table S3, Tab 1). To determine if CAVIN1 directly influences hnRNPK localisation or acts by neutralizing non‐caveolar CAV1, we ectopically expressed CAV1 in two cell lines that naturally lack CAV1 and CAVIN1, namely the androgen‐sensitive prostate cancer cell line LNCaP, and human embryonic kidney HEK293 cells. While hnRNPK was found to be entirely localised in the nucleus in LNCaP‐CONT and HEK293‐CONT cells, expression of CAV1 alone caused the translocation of hnRNPK to the MVB (Figures 2C and 2D, S2, and S3). This was further solidified by co‐localisation analysis between hnRNPK and CD9 for each condition (Figures 2B–2D, Table S3, Tab 1). In contrast, expression of CAVIN1 alone reduced hnRNPK expression in the nucleus but did not induce MVB localisation in LNCaP cells (Figure S2). These results suggest that non‐caveolar CAV1 drives MVB hnRNPK localisation, while CAVIN1 neutralises CAV1 action.

### 3.3 hnRNPK modulates PC3 miRNA composition and EV osteoclastogenesis activity

**FIGURE 3:**
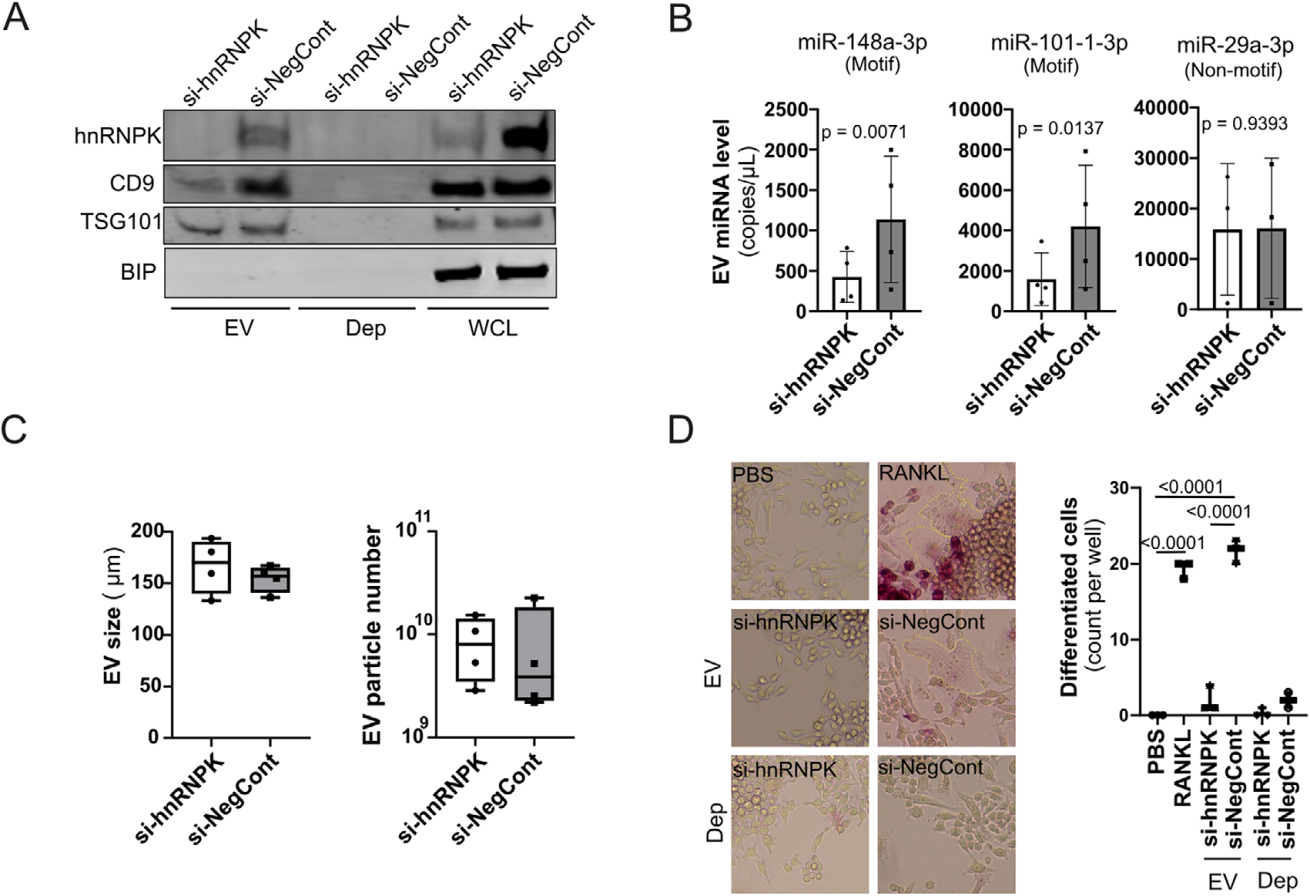
Depleting hnRNPK reduces secretion of motif‐containing miRNA and osteoclastogenic potential of PC3‐derived EVs. **(A)** Representative western immunoblotting of hnRNPK, CD9, TSG101 and BIP in EVs from cells treated with hnRNPK targeting siRNA (si‐hnRNPK) or non‐targeting control (si‐NegCont) siRNA confirms hnRNPK knockdown efficiency and EV purity. Note that 10 μg of EV and whole cell lysate (WCL), and equal volume EV‐depleted media were loaded to each well. (**B)** EV content of miR‐148a‐3p, miR‐101‐3p and miR‐29a‐3p from si‐NegCont and si‐hnRNPK treated cells measured by ddPCR. (**C)** EV number and size analysis of EVs extracted from si‐NegCont and si‐hnRNPK treated cells by NanoSight. All error bars represent standard deviation (*n* = 4). All statistical tests used were two‐sided and paired. (**D)** Representative images of RAW264.7 cells treated with si‐NegCont and si‐hnRNPK EVs, EV‐depleted media (dep) or controls (40 ng/mL RANKL positive differentiation control, or PBS negative control) for a total of 5 days, and evaluated for TRAP and multinuclear status *(left)*. Multinucleated and TRAP resistant cells were counted for each condition and shown in a box‐and‐whisker plot. *n* = 3, and *p*‐value calculated using a two‐sided ANOVA

To establish a role for hnRNPK in EV miRNA composition, we next tested if hnRNPK is required for PC3 loading of AsUGnA motif‐containing miRNA into EVs. We conducted hnRNPK knockdown in PC3 cells using hnRNPK‐targeting siRNAs (si‐hnRNPK, SMARTpool) or non‐targeting siRNA (si‐NegCont). Knockdown efficacy and EV purity were confirmed by western immunoblotting for hnRNPK, EV markers CD9 and TSG101, and the endoplasmic reticulum marker binding immunoglobulin protein (BIP) (Figure 3A).

The effect of si‐hnRNPK on the EV composition of selected miRNAs was evaluated using ddPCR. For this analysis, we chose two motif‐containing miRNA (miR‐148a‐3p and miR‐101‐1‐3p) and 1 miRNA without motif (miR‐29a‐3p). In agreement with a role for AsUGnA motif in hnRNPK‐mediated EV loading, motif‐containing miR‐148a‐3p and miR‐101‐1‐3p were downregulated in EVs when cellular hnRNPK levels are reduced, but non‐motif miRNA miR‐29a‐3p was not affected (Figure 3B). Assessment of EV number and size by nanoparticle tracking analysis showed no impact of hnRNPK knockdown (Figure 3C), indicating that hnRNPK is not required for EV biogenesis.

To assess the functional impact of si‐hnRNPK EVs, we made use of our previously established assay of PC3 EV‐induced RAW264.7 cell osteoclastogenesis.^4^ Osteoclast formation and activity are crucial in the pre‐metastatic niche in bone,^34^ and EV‐delivered miRNAs have been implicated in bone remodelling.^35^ Indeed, we previously showed that PC3 EVs alone induced osteoclastogenesis of the model RAW264.7 cell line to a similar extent as the positive control RANKL,^4^ which was replicated by the negative control siRNA condition in the current experiment, si‐NegCont EV (Figure 3C). Strikingly, si‐hnRNPK EVs induced minimal osteoclastogenesis, comparable to EV‐depleted media from si‐NegCont (Figure 3C). This assay suggests that hnRNPK is required for EV‐mediated osteoclastogenesis.

Taken together, the hnRNPK knockdown experiments establish a functional pro‐metastatic role for hnRNPK positive EVs, potentially via targeted recruitment of specific miRNAs.

### 3.4 hnRNPK directly binds miR‐148a‐3p at the conserved AsUGnA motif

While hnRNPK is known to bind messenger RNA (mRNA), there has only been a single report on miRNA binding,^36^ and no specific RNA motif apart from a short poly‐C region.^37^ Therefore, we conducted a series of experiments to evaluate hnRNPK‐miRNA interactions. We chose miR‐148‐3p as proof of concept since it contains the AsUGnA motif, and we had previously validated this miRNA in PC3 cells and EV by qRT‐PCR.^4^

**FIGURE 4:**
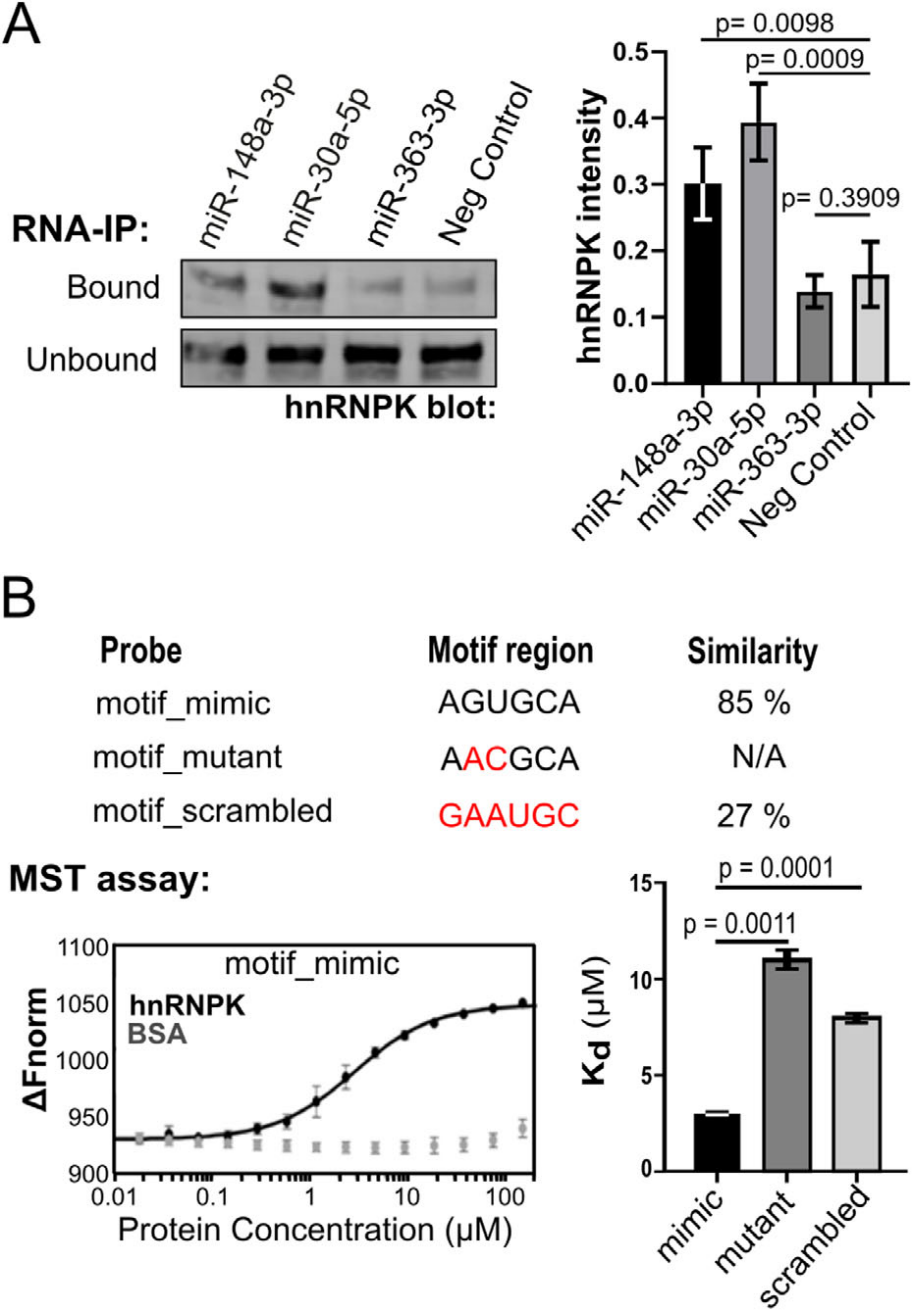
hnRNPK directly binds to motif‐containing miRNA to regulate EV export. **(A**) Representative hnRNPK immunoblotting RNA‐immunoprecipitation (RNA‐IP) experiments using either biotinylated miR‐148a‐3p, miR‐30a‐5p, miR‐363‐3p or scrambled miR‐148a‐3p (Neg. Control). Supernatants (20% of total) from the pulldowns were probed to confirm the presence of hnRNPK in the cell lysate. Band intensity was quantitated from four separate experiments, normalised across the replicate and compared to negative control condition using parametric *t*‐test. (**B**) Microscale thermophoresis (MST) assay was used to assess in vitro binding between recombinant hnRNPK at increasing doses, and miR‐148a‐3p and two motif mutants. *Top*, similarity between probe sequence and putative hnRNPK‐binding motif was compared for each probe, where similarity scores (%) are reported for matches in the tested motif region (N/A, if no match was made in motif region). *Left*, example dose response curve shows increased binding affinity (Δ Fnorm, normalised change in fluorescence) with increasing concentrations of hnRNPK, but not BSA (control). *Right*, dissociation constants (K_d_) calculated from the MST dose response curves (*n* = 3, ± standard deviation) show hnRNPK binding affinity to miR‐148a‐3p (motif_mimic), miR‐148a‐3p with ‘mutated motif’ (motif_mutant) and with a scrambled motif (motif_scrambled), with lower K_d_ indicating stronger binding

Firstly, biotinylated miRNA mimics were used to pull down binding proteins from PC3‐CONT (CAV1+/CAVIN‐) cell lysate. For each biological replicate, the same cell lysate was split across four tubes containing biotin‐miR‐148a‐3p (miR‐148a‐3p, motif‐containing), biotin‐miR‐30a‐5p (miR‐30a‐5p, motif‐containing), biotin‐miR‐363‐3p (miR‐363‐3p, non‐motif containing) and biotin‐scrambled miRNA (negative control), respectively. Then, streptavidin‐coated beads were used to isolate the biotin‐miR bound proteins. hnRNPK binding to these target miRNA was determined by immunoblotting and quantitation of the band intensity. A fraction of the supernatant (unbound) after pulldown was also loaded as a control to confirm the presence of hnRNPK in the lysate. As shown in Figure 4A, hnRNPK was detected in motif‐containing miR‐148a‐3p and miR‐30a‐5p pulldown but not in the miR‐363‐3p or control pulldown, demonstrating the ability of hnRNPK to specifically bind motif‐containing miRNAs in the cellular context.

To further confirm direct binding, we performed in vitro microscale thermophoresis assay using recombinant hnRNPK, and 5′ 6‐FAM tagged miR‐148a‐3p. Microscale thermophoresis uses fluorescence to monitor the movement of a molecule in response to an applied temperature gradient. Compared to BSA (binding control), the increased binding between miR148a‐3p with increasing concentrations of hnRNPK (dose response) was observed by increasing delta normalised fluorescence (ΔFnorm, Figure 4B). To determine if the RNA motif AsUGnA mediates hnRNPK binding for miR‐148a‐3p, dissociation constants were calculated for the binding between hnRNPK and miR‐148a‐3p mimic and miR‐148a‐3p mutant with substitutions at positions 2 and 3 of the motif (motif_mutant), or a scrambled motif region (motif_scrambled). The higher K_d_ observed for the mutant and scrambled miRNAs in Figure 4B show that changes to the AsUGnA motif within miR‐148a‐3p sequence significantly reduced binding strength, indicating that this motif participates in hnRNPK binding to miR‐148a‐3p. Taken together, these data confirm the direct interaction between hnRNPK and miR‐148a‐3p via the AsUGnA motif.

### 3.5 hnRNPK co‐localises with selectively exported miRNAs in MVBs

**FIGURE 5:**
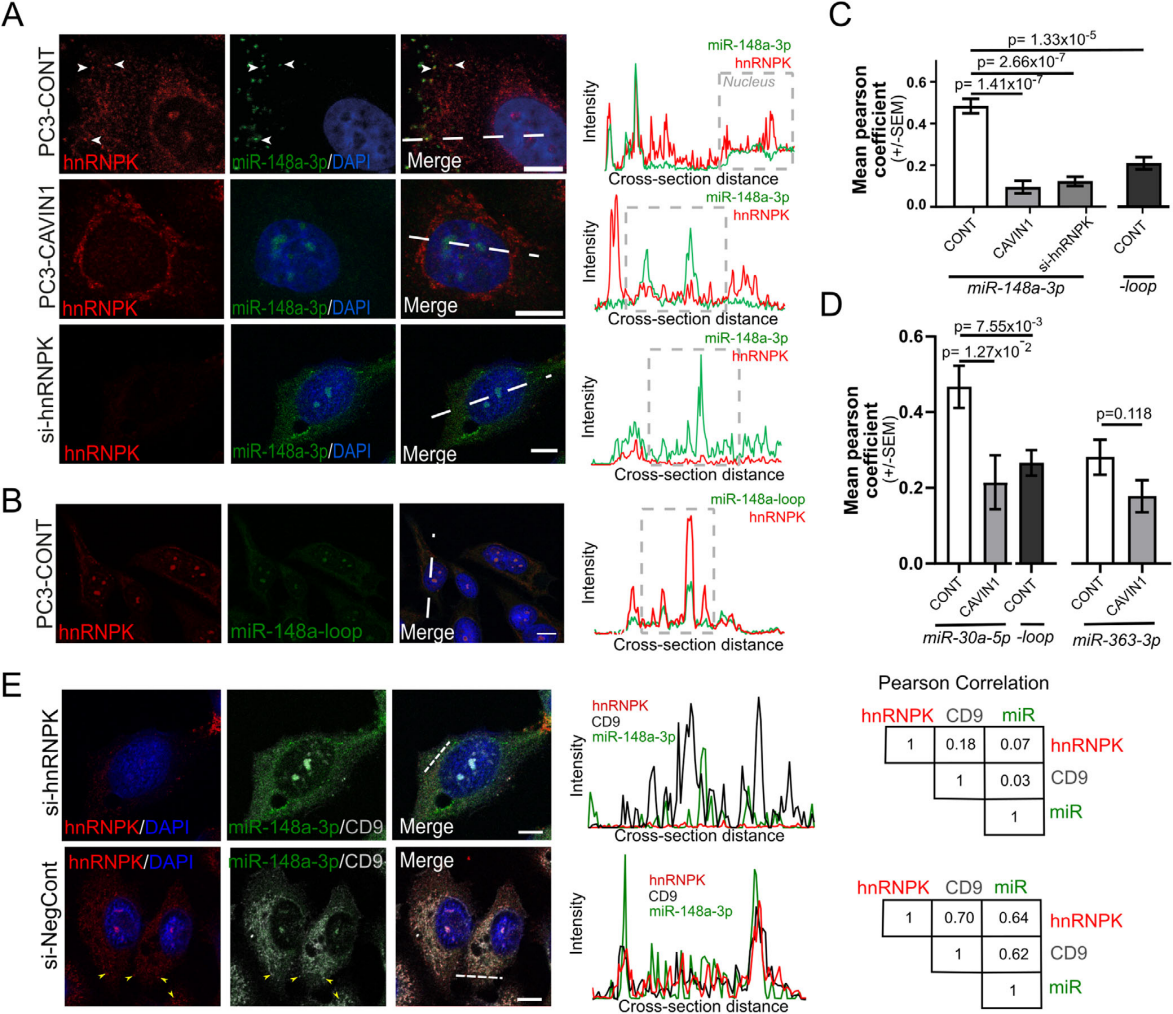
hnRNPK co‐localises with mature motif‐containing miRNA in PC3‐CONT cells. Fluorescent probes (green) targeting the 3 prime arm of miR‐148a (miR‐148a‐3p, **A**) or the precursor‐specific loop region of miR‐148a (miR‐148a‐loop, **B**) in PC3 cells relative to hnRNPK (red). DAPI staining shows the nucleus in blue. Profile plots (right) display hnRNPK intensity relative to miRNA intensity for a cross‐section of the cell (nuclear region boxed). Scale bars are 10μm. **(C and D)** Quantitative co‐localisation analysis was performed in PC3 cells between hnRNPK and miR‐148a (C), miR‐30a (D), and miR‐363‐3p (D) probes. Each bar represents Pearson correlation coefficients from 10 regions of interest for each condition, from three to four biological replicates, two‐three cells per replicate (individual data points supplied in Table S3, Tab 2). A two‐sided unpaired Mann‐Whitney test was used to determine significance. (**E)** Localisation of hnRNPK, miR‐148a‐3p and exosome marker (CD9) were visualised and analysed by profile plot and Pearson correlation between profiles. This is shown in PC3 cells treated with hnRNPK‐targeting (si‐hnRNPK) or non‐targeting siRNA (si‐NegCont)

Having confirmed direct binding of hnRNPK with the AsUGnA motif in miR‐148a‐3p, we next examined the localisation of hnRNPK and motif‐containing miRNA in the PC3 cell model. miRNA in situ hybridisation (miR‐ISH) using fluorescent Cy5‐conjugated antagomiRs was coupled with indirect immunofluorescence using hnRNPK antibody to detect hnRNPK. Representative images for each condition are shown in Figure 5 with qualitative profile plot highlighting the spatial detection of hnRNPK and target miRNA. Quantitative co‐localisation analysis on 10 regions per condition was used to quantitate co‐localisation between miR‐148a probes and hnRNPK in each condition (Figure 5C, Table S3, Tab 2).

As shown in Figure 5A, miR‐148a‐3p co‐localised with hnRNPK at distinct cytoplasmic puncta in PC3‐CONT cells, but not in PC3‐CAVIN1 cells. On the other hand, in the absence of hnRNPK (si‐hnRNPK, Figure 5A), miR‐148a‐3p localised to nuclear bodies. This result suggests that nuclear bodies are the default localisation for miRNA when not bound by hnRNPK and that hnRNPK does not interact with the target miRNA in PC3 CAVIN1 cells, potentially because of differential compartmentalisation and/or post‐translational modification. As precursor miR‐148a possesses the validated sequence motif, we also evaluated its co‐localisation using a loop region probe. We observed co‐localisation of hnRNPK and loop probe (specific to precursor form) in nuclear bodies but not in cytoplasmic puncta (Figure 5C). This result suggests that mature miRNA are most likely the target of hnRNPK‐mediated exosomal sorting.

To supplement this observation, we assessed hnRNPK co‐localisation with an additional motif‐containing miRNA (miR‐30a‐5p), as well as non‐motif containing miRNA (miR‐363‐3p) and a non‐targeting scrambled control (Figure 5D, S4, Table S3). Consistent with the miR‐148a‐3p results, miR‐30a‐5p (but not precursor miR‐30a) co‐localised with hnRNPK in cytoplasmic puncta in PC3‐CONT cells, whereas miR‐363‐3p displayed a diffuse cytoplasmic signal not specific to hnRNPK (Figure S4). The co‐localisation observed between miR‐30a‐5p and hnRNPK in cytoplasmic puncta was lost in PC3‐CAVIN1 cells, but co‐location at the nuclear bodies was maintained (Figure S4). These results confirm the specific interaction between hnRNPK and mature motif‐containing miRNA to cytoplasmic puncta.

Lastly, we tested the hypothesis that hnRNPK‐miR‐148a‐3p interaction occurs at forming EVs of endosomal origin (exosomes) by co‐localisation analysis of both hnRNPK and miR‐148a‐3p with the MVB marker CD9 (Figure 5E). Co‐localisation of hnRNPK, miR‐148a‐3p and CD9 was assessed by profile plot and Pearson correlation in PC3 cells which had been treated with hnRNPK siRNA (si‐hnRNPK) or non‐targeting control (si‐NegCont). As shown in Figure 5E, high correlation of hnRNPK, miR‐148a‐3p and CD9 was observed in si‐NegCont PC3 cells (*r* = 0.62–0.7), while no co‐localisation was observed between these molecules after treatment with si‐hnRNPK (*r* = 0.03–0.18), indicating that hnRNPK is required for localisation of miR‐148a‐3p to the forming exosome.

### 3.6 Membrane lipid environment dictates hnRNPK localisation

**FIGURE 6:**
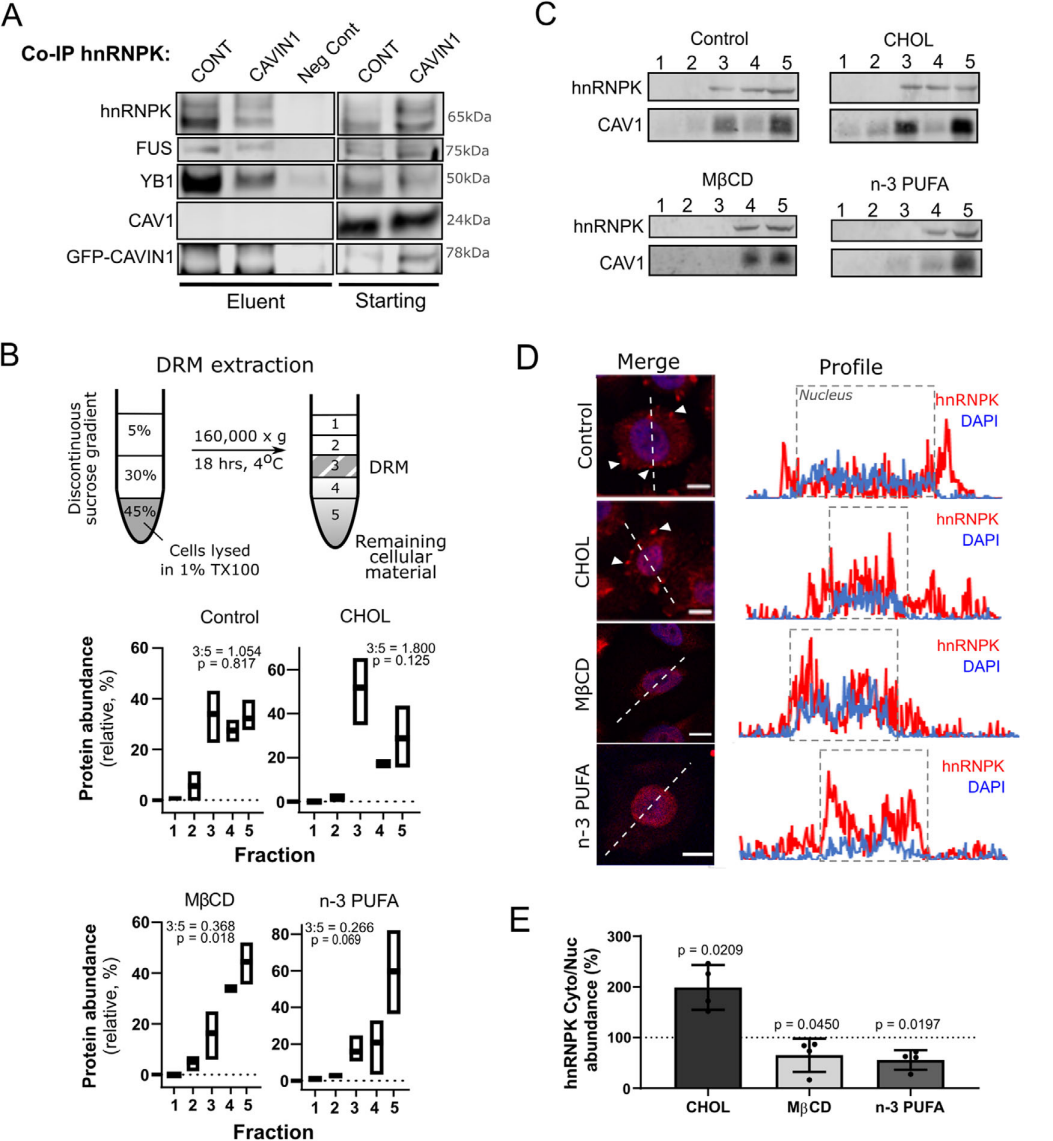
Remodelling of membrane raft environments regulates hnRNPK localisation. **(A)** Western blot following co‐immunoprecipitation to assess hnRNPK binding partners in PC3‐CONT (CONT) and ‐CAVIN1 (CAVIN1) cells. Eluent and starting lysate were blotted for hnRNPK, as well as known hnRNPK binding partners, FUS and YB1, and potential binders CAV1 and CAVIN1. Non‐targeting IgG was used as a negative control (Neg Cont) for the immunoprecipitation experiment using PC3‐CONT lysate. (**B‐E)** PC3 cells were exposed to 500μM MβCD, 50μM cholesterol (Chol), or n‐3 PUFA (30μM DHA: 45μM EPA) in LPDS/RPMI1640 for 24 h. Cells were exposed to LPDS/RPMI1640 as a non‐treatment control (termed Control). (**B)** Sucrose gradient separation of detergent resistant membranes (DRMs, fraction 3) from cellular membranes (fraction 4 and 5) of PC3 cells. The effect of CHOL, MβCD and n‐PUFA on DRM was measured by relative protein abundance of each fraction (mean ± SD, fraction abundance relative to total protein). Bar depicts max‐mix range and mean for three biological replicates. Note that 3:5 ratio was calculated comparing mean abundance of fraction 3 and 5, and *p* value compared abundance between 3 and 5. (**C)** DRM fractions were immunoblotted for hnRNPK and CAV1 (membrane raft marker). Equal volume was loaded into each well. (**D)** Localisation of hnRNPK (red) and DAPI (blue) following exposure to lipid manipulation reagent. Scale bar is 10 μm. Profile plots (right) highlight the overlap between hnRNPK and DAPI signal, with the nuclear regions boxed. For additional replicates (*n* = 3), see Figure S5. (**E)** Cytoplasmic‐to‐nuclear hnRNPK ratio compared across the different manipulations, relative to non‐treatment control (100%). Error bars represent standard deviation (*n* = 4 per condition). Paired *t*‐test compares each condition to negative control. Figure S5 displays western immunoblot underpinning this figure, and quantitation available in Table S4

CAV1 binding to hnRNPKA2/B1 was recently reported as a mechanism of loading miRNA to microvesicles in lung epithelial cells.^38^ While we observed that CAV1 is indeed released in PC3 EV via the endosomal‐pathway,^39^ its release is not altered in PC3‐CAVIN1 cells.^4^ To test the possibility that CAV1 binds to hnRNPK in the PC3 cell model, we conducted a coimmunoprecipitation experiment to find hnRNPK binding partners in both PC3‐CONT and PC3‐CAVIN1 whole cell lysates. Western blotting of the eluent identified known hnRNPK binding partners; FUS and YB1, but not CAV1 or CAVIN1 (Figure 6A). These results indicate non‐caveolar CAV1 regulates hnRNPK localisation via indirect mechanisms.

As CAV1 and CAVIN1 regulate the formation of caveolae, we speculated that hnRNPK localisation may be associated with membrane raft structures. To test this hypothesis, we monitored hnRNPK localisation after exposure to compounds which target membrane raft formation, namely cholesterol, methyl‐β‐clycodextrin (MβCD) and omega‐3 polyunsaturated fatty acids (n‐3 PUFA). While addition of cholesterol induces tight clustering of sphingolipids to form membrane rafts, addition of bulky n‐3 PUFA destabilises this clustering effect by displacing cholesterol and decreasing molecular order.^14^, ^40^

PC3 cells were treated with cholesterol (CHOL), the cholesterol‐chelating agent Methyl‐β‐cyclodextrin (MβCD) or n‐3 PUFA (EPA and DHA) for 24 h. Membrane raft fraction was collected by the DRM method involving detergent extraction and sucrose gradient (Figure 6B). DRM protein abundance (Figure 6B; TableS4, Tab 1) and immunoblotting for CAV1 as a known membrane raft protein confirmed successful DRM floatation to fraction 3, where hnRNPK was also detected in control treatment (Figure 6C). As expected, MβCD and n‐3 PUFA treatment disrupted membrane rafts, as seen by loss of CAV1 from fraction 3 and reduction of DRM protein. In agreement with our hypothesis, hnRNPK was also lost from fraction 3 under the same conditions (Figure 6C). Furthermore, relative DRM protein content across the conditions shows the same trend (Figure 6B; Table S4, Tab 1), indicating that hnRNPK is regulated with other raft proteins in PC3 cells.

We then assessed whether hnRNPK localisation is altered by raft disruption using immunofluorescent microscopy (Figure 6D). Extranuclear hnRNPK (red) was observed in control and cholesterol treatments (arrows in Figure 6D) but tends to be reduced upon raft disruption by MβCD and n‐3 PUFA, with increased localisation to nuclear regions (blue, region boxed). To quantify this trend, we performed an independent experiment where we extracted cytoplasmic and nuclear fractions from exposed cells, and measured hnRNPK signal in each compartment via quantitative immunoblotting (Figure 6E). Cytoplasmic‐to‐nuclear ratios were compared to non‐treatment control to determine deviation from the baseline cytoplasmic abundance. Cholesterol exposure increased hnRNPK cytoplasmic signal compared to nuclear compartment (Figure 6E, Table S4, Tab 2), consistent with increased membrane raft formation. Conversely, disruption of membrane rafts via n‐3 PUFA or MβCD resulted in reduced cytoplasmic hnRNPK content and redistribution to the nuclear compartment. These data confirm aberrant hnRNPK localisation is induced by membrane raft formation driven by non‐caveolar CAV1, where remodelling by treatment or caveolar formation returns hnRNPK to canonical nuclear localisation.

### 3.7 Elevated EV hnRNPK detected in body fluids from metastatic prostate and colorectal cancers

**FIGURE 7:**
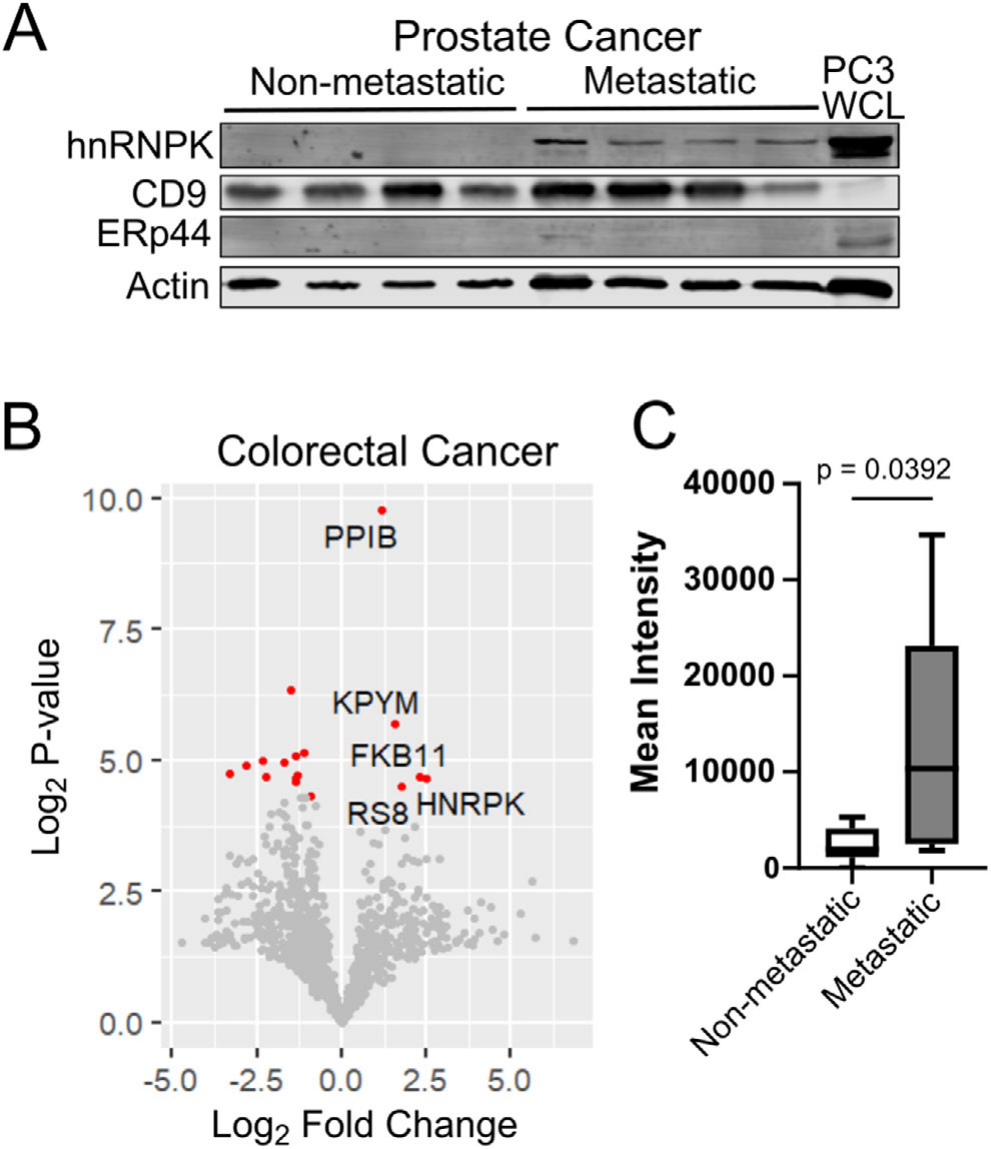
EV hnRNPK is elevated in body fluids from metastatic prostate and colorectal cancer. **(A)** EVs were prepared from seminal plasma samples from patients with clinically localised prostate cancer and metastatic prostate cancer. Extracts (15 μg EV protein or PC3 whole cell lysate) were immunoblotted for hnRNPK, EV markers CD9 and actin, and cellular marker ERp44. (**B)** SWATH data for plasma EV proteomics derived from CRC patients with either metastatic (Grade III and IV, *n* = 8) or non‐metastatic (Grade I and II, *n* = 8) disease. Log_2_ fold changes (log2FC) were calculated for all proteins detected in CRC EVs, comparing low‐ to high‐grade protein levels, and *p*‐value calculated using a two‐sided unpaired *t*‐test. All significant proteins (*p*‐value < 0.05) are shown in red, and proteins increased in metastatic disease labelled. For normalised intensity and processed data, see Table S5. (**C)** Mean normalised protein intensities for plasma EV hnRNPK for non‐metastatic and metastatic CRC

Finally, we went on to measure hnRNPK EV protein in cancer biofluids. We made use of a limited cohort of prostate cancer patients with long‐term follow‐up. Seminal plasma EVs collected by ultracentrifugation were immunoblotted for hnRNPK from four localised prostate cancer and four metastatic prostate cancer cases. A cellular marker (ERp44) and EV markers (CD9 and β‐actin) were used to assess cellular contamination and confirm EV isolation. In this small cohort, all four metastatic cases displayed elevated levels of EV hnRNPK, with no detectable signal observed in the non‐metastatic samples (Figure 7A).

To further extend this finding, we next examined plasma EVs from a cohort of colorectal cancer patients, as CAV1 has been previously associated with metastatic CRC,^41^, ^42^, ^43^ and hnRNPK has been detected in both EV and membrane raft proteomes of colorectal cancer.^44^, ^45^, ^46^ Unbiased proteomics using SWATH‐MS was conducted on plasma EVs from a cohort of 16 CRC patients: eight early (Grade I and II) and eight metastatic (Grade III and IV). Of the 1150 proteins measured, 16 proteins were significantly different between early and metastatic disease (Figure 7B, Table S5). Only five EV proteins were significantly higher in metastatic CRC, with hnRNPK having the highest fold‐change (log2FC = 2.47, *p*‐val = 0.039). The boxplot in Figure 7C shows the normalised intensity of hnRNPK levels in plasma EVs from early versus metastatic CRC. Taken together, these limited clinical cohort studies conducted using independent methods suggest a potential role for EV hnRNPK in metastatic disease.

## 4 DISCUSSION

Recent studies highlight key roles of EVs in multiple steps of cancer progression, from metastatic niche establishment to immune suppression.^7^, ^47^ Given their pleiotropic actions, inhibiting the release of metastasis‐promoting EVs would be an attractive approach, if the mechanisms of this EV cargo loading were known and targetable. Initial research on cancer exosomes focused on the proteomic cargo, but recent studies highlight key roles for exosomal miRNA in cancer.^48^ Furthermore, while many studies consider the importance of lipid composition in EV biogenesis, this is the first study to define the molecular components of membrane lipid‐mediated selective EV miRNA loading. Specifically, this study reveals a mechanistic link between non‐caveolar CAV1‐driven membrane remodelling and exosomal miRNA composition, through motif‐guided miRNA recruitment via hnRNPK protein redistribution. The functional role for hnRNPK in PC3 EV‐mediated osteoclastogenesis and EV hnRNPK detection in metastatic prostate and colorectal cancers implicate EV hnRNPK as a potential biomarker for metastatic cancer. Importantly, we show that raft disruption by MβCD or n‐3 PUFA effectively reduced hnRNPK cytoplasmic localisation, opening the door for potential metastasis chemoprevention.

This study utilised non‐caveolar CAV1 in aggressive prostate cancer cells as a model system to investigate the molecular mechanisms of miRNA loading to EVs. CAV1 primarily resides in plasma membrane caveolae in the presence of the co‐factor protein CAVIN1.^1^, ^49^ In the absence of CAVIN1, non‐caveolar CAV1 is internalised for degradation via endolysosomes.^1^, ^49^ This continual endocytosis of non‐caveolar CAV1 also leads to broader membrane remodelling, impacting MVB and exosome composition. Indeed, recent studies implicate intracellular CAV1 in regulating various organelle compartments, with particular emphasis on roles in autophagosomes and cancer.^39^, ^50^, ^51^ The premise that membrane remodelling drives exosomal miRNA sorting is consistent with our prior observation that CAVIN1 attenuates non‐caveolar CAV1 in prostate cancer but CAVIN1 itself was not detected in PC3 EVs.^2^

In addition to CAV1, other metastasis‐promoting genes like Ras family proteins and tetraspanins also impact membrane composition,^52^, ^53^, ^54^ hence, membrane remodelling appears to be a common mechanism underlying release of pro‐metastatic EVs in cancers. The role of membrane raft proteome in mediating cholesterol‐associated metastasis was previously highlighted in our prostate cancer xenograft hypercholesterolemic diet study,^55^ which identified a cholesterol‐responsive protein, IQGAP1, that mediates cell migration and invasion and was essential for hypercholesterolemia‐induced metastasis in vivo.^55^ While cholesterol is known to regulate gene expression via SREBP and LXR, cholesterol treatment did not increase IQGAP1 transcription in prostate cancer cells, but rather increased membrane raft IQGAP1,^55^ presumably stabilizing the protein. Together with the new data showing membrane rafts regulate hnRNPK translocation and EV miRNA, membrane remodelling appears to activate multiple metastasis‐promoting pathways. This process could be targeted in novel metastasis chemoprevention.

The classic gene silencing role of miRNAs involves direct interactions with Argonaute proteins (AGO) and formation of miRNA‐induced silencing complex (miRISC) leading to translational repression and/or degradation of target mRNAs.^56^ miRISCs have been detected in several subcellular compartments in the cytoplasm (P‐bodies, stress granules, MVB, ER) as well as in the nucleus.^57^ The importance of subcellular location of specific miRISC is beginning to be appreciated.^57^ In one recent study, a paraspeckle protein SFPQ (splicing factor proline/glutamine rich) has been reported as influencing mRNA‐miRNA targeting via RNA sequence motifs and AGO2 interactions in both nucleus and cytoplasm.^58^ However, it is still unknown if hnRNPK could itself interact with miRISC and play a role in miRNA regulation in the nucleus of non‐malignant cells. As reported by Treiber et al, the various members of the hnRNP family also seem to participate in miRNA biogenesis^59^; however, their exact role and whether miRISC is involved are yet to be determined. It is tempting to suggest that hnRNPs involved in biogenesis simply becomes mis‐localised under certain stimuli, leading to altered miRNA loading to EVs. This hypothesis should be evaluated in future studies.

This study further establishes the hnRNP family as EV miRNA chaperones, following recent reports for hnRNPA2B1 and hnRNPQ.^27^, ^28^ Lee et al recently reported direct interaction with phosphorylated CAV1 mediates hnRNPKA2B1 EV recruitment.^38^ In PC3 cells, we did not detect direct interaction between hnRNPK and CAV1, but demonstrated non‐caveolar CAV1‐induced membrane raft‐mediated hnRNPK translocation to MVB/EV. We speculate that different mechanisms regulate the EV targeting of different hnRNP family members, loading a different miRNA repertoire to EVs. Future studies should investigate the differential regulatory mechanisms of hnRNP family members’ subcellular localisation and miRNA sorting.

hnRNPK shuttles between nuclear and cytoplasmic stress granules in healthy cells, regulated by phosphorylation.^60^, ^61^ It is predominantly nuclear in non‐malignant cells (Figure 4C ^62^), but reported to be overexpressed in various cancers with an increase in cytoplasmic localisation.^29^, ^63^ This change in subcellular localisation is proposed to be a major regulator in hnRNPK pro‐oncogenic functions over its canonical healthy nuclear function. This study provides the first evidence that cytoplasmic hnRNPK co‐localises with MVB and participates in motif‐based miRNA loading to forming exosomes. While we demonstrate the raft‐dependency of hnRNPK translocation, further work is required to establish how hnRNPK interacts with membrane rafts in a regulated manner.

In summary, our study confirms hnRNPK as a motif‐dependent exosomal miRNA‐sorting protein, that is, regulated by membrane rafts and provides initial evidence for elevated EV hnRNPK in metastatic cancers. Raft disruption by cholesterol depletion or n‐3 PUFA treatment is a potential approach to attenuate metastatic EV production. While we have focused on implications in cancer, extra‐nuclear hnRNPK has been reported to play a role in several other biological processes, including axonal outgrowth^64^ via local axonal mRNA translation^65^ as well as regulation of dendritic spine morphology.^66^ Hence, membrane remodelling may have broader roles in RNA subcellular localisation and release.

## CONFLICT OF INTERESTS

The authors have no competing financial interests to declare.

## AUTHOR CONTRIBUTIONS

Michelle M. Hill and Alexandre S. Cristino conceived and supervised the project. Harley Robinson, Archa H. Fox, Nicole Cloonan, Renée S. Richards, Carlos Salomon, Alexandre S. Cristino and Michelle M. Hill contributed to experimental design. David Margolin, Li Li, Archa H. Fox and Robert A. Gardiner contributed clinical cohort. Harley Robinson, Jayde E. Ruelcke, Amanda Lewis, Vandhana Bharti, Shivangi Wani and Andrew Lai performed laboratory research. Harley Robinson, Nicole Cloonan and Alexandre S. Cristino performed computational analyses. Charles S. Bond, Archa H. Fox, Nicole Cloonan, Robert A. Gardiner, Robert G. Parton, Alexandre S. Cristino and Michelle M. Hill contributed to data interpretation. Harley Robinson, Alexandre S. Cristino and Michelle M. Hill wrote the manuscript. All authors approved the manuscript.

## Supporting information

Supporting materials and methods: SWATH library generation and sample processing.
Figure S1: hnRNPK translocates between MVB in PC3‐CONT cells to mitochondrial and ER regions in PC3‐CAVIN1 cells, related to Figure 2. CD9 (MVB marker), ERp44 (ER marker) and VDAC4 (mitochondrial marker) localisation (green) relative to hnRNPK (red) in PC3‐CONT (A) and PC3‐CAVIN1 (B) cells. DAPI (blue) displays nuclear localisation. Images taken from two to four biological replicates. Scale bars are 10 μm. Boxed areas in top panel indicate ROI for co‐localisation analysis. Data retrieved from analysis are available in Table S3.
Figure S2: hnRNPK only translocates to MVB compartments in CAV1 expressing LNCaP cells, related to Figure 2. Images show hnRNPK (red) localisation in LNCaP‐CONT (A), LNCaP‐CAV1 (B) and LNCaP‐CAVIN1 (C) cell lines, relative to CD9 (green) and DAPI (blue) staining. Images were taken from three biological replicates. Scale bar denotes 10 μm. Boxed regions in composite (merge) indicate ROIs for co‐localisation analysis. Data retrieved from analysis are available in Table S3.
Figure S3: hnRNPK only translocates to MVB compartments in CAV1 expressing HEK293 cells, related to Figure 2. Additional images showing hnRNPK (red) and CD9 (green) localisation in HEK293‐CONT (A) and HEK293‐CAV1 (B) cell lines, and DAPI (blue) staining. Images were taken from three biological replicates. Scale bar denotes 10 μm. Boxed regions in composite (merge) indicate ROIs for co‐localisation analysis. Data retrieved from analysis are available in Table S3.
Figure S4: Motif‐containing mature miR‐30a co‐localised with hnRNPK in PC3‐CONT cells, related to Figure 5. Mature motif‐containing miR‐30a‐5p, precursor miR‐30a (miR‐30a‐loop), a motif‐absent miR‐363‐3p and a scrambled miRNA (green) control were visualised using miR‐ISH and co‐localised with hnRNPK (red) in PC3‐CONT and PC3‐CAVIN1 cells. Ten ROIs were used for co‐localisation analysis (right), where applicable. Data retrieved from analysis are available in Table S3.
Figure S5: Membrane raft alterations modify hnRNPK localisation in PC3 cells, related to Figure 6. A) hnRNPK (red) and DAPI (blue) were visualised after exposure of PC3 cells to 500μM MβCD, 50μM cholesterol (CHOL), or n‐3 PUFA (30μM DHA: 45μM EPA) in LPDS/RPMI1640 for 24 h across three biological replicates. B) Western immunoblot comparing nuclear and cytoplasmic signal for hnRNPK, DDX5 (nuclear marker), HSP90 (cytoplasmic marker) and actin (loading control). Note that 10 μg protein loaded into each well. Quantitation of each band used for analysis is available in Table S4 for all replicates.

Table S1: Patient information tables, related to Methods. Prostate cancer patient information (Tab 1) and colorectal cancer patient information (Tab 2).

Table S2: DESeq2 results for cellular miRNA (Tab 1), EVs (Tab 2), and the associated classification analysis (Tab 3), motif enrichment (Tab 4), motif scanning (Tab 5) and pathway enrichment analysis (Tab 6, PANTHER and Reactome analyses) related to Figure 1.

Table S3: Co‐localisation analysis between hnRNPK and CD9 (Tab 1), and hnRNPK and miRNA targets (Tab 2), related to Figures 2 and 5.

Table S4: Quantitative analyses for raft disruption treatments for DRM protein abundance (Tab 1) and hnRNPK level in cytoplasmic and nuclear fractions (Tab 2), related to Figure 6.

Table S5: SWATH data from serum EVs extracted from early and metastatic CRC patients, relating to Figure 7

Table S6: Complete list of oligonucleotides used in this paper. Each custom made oligonucleotide was purchased from Integrated DNA technologies (IDT).

## Data Availability

The RNA sequencing data have been deposited to the Gene Expression Omnibus (GEO) under the accession number GSE109356.

## References

[1] Hill MM , Bastiani M , Luetterforst R , et al. PTRF‐cavin, a conserved cytoplasmic protein required for caveola formation and function. Cell. 2008;132(1):113–124. 18191225 10.1016/j.cell.2007.11.042 PMC2265257

[2] Moon H , Lee CS , Inder KL , et al. PTRF/cavin‐1 neutralizes non‐caveolar caveolin‐1 microdomains in prostate cancer. Oncogene. 2014;33(27):3561–3570. 23934189 10.1038/onc.2013.315

[3] Inder KL , Zheng YZ , Davis MJ , et al. Expression of PTRF in PC‐3 cells modulates cholesterol dynamics and the actin cytoskeleton impacting secretion pathways. Mol Cell Proteomics. 2012;11(2):M111.012245. 10.1074/mcp.M111.012245 PMC3277761 22030351

[4] Inder KL , Ruelcke JE , Petelin L , et al. Cavin‐1/PTRF alters prostate cancer cell‐derived extracellular vesicle content and internalization to attenuate extracellular vesicle‐mediated osteoclastogenesis and osteoblast proliferation. J Extracell Vesicles. 2014;3:23784. 10.3402/jev.v3.23784 PMC4072912 25018864

[5] Vlassov AV , Magdaleno S , Setterquist R , Conrad R . Exosomes: current knowledge of their composition, biological functions, and diagnostic and therapeutic potentials. Biochim Biophys Acta. 2012;1820(7):940–948. 22503788 10.1016/j.bbagen.2012.03.017

[6] Witwer KW , Théry C . Extracellular vesicles or exosomes? On primacy, precision, and popularity influencing a choice of nomenclature. J Extracellular Vesicles. 2019;8(1):1648167. 31489144 10.1080/20013078.2019.1648167 PMC6711079

[7] Becker A , Thakur BK , Weiss JM , Kim HS , Peinado H , Lyden D . Extracellular vesicles in cancer: cell‐to‐cell mediators of metastasis. Cancer Cell. 2016;30(6):836–848. 27960084 10.1016/j.ccell.2016.10.009 PMC5157696

[8] Kim Y‐K . Extracellular microRNAs as biomarkers in human disease. Chonnam Med J. 2015;51(2):51–57. 26306299 10.4068/cmj.2015.51.2.51 PMC4543150

[9] Guo H , Ingolia NT , Weissman JS , Bartel DP . Mammalian microRNAs predominantly act to decrease target mRNA levels. Nature. 2010;466(7308):835–840. 20703300 10.1038/nature09267 PMC2990499

[10] Andreu Z , Yáñez‐Mó M . Tetraspanins in extracellular vesicle formation and function. Front Immunol. 2014;5:442–442. 25278937 10.3389/fimmu.2014.00442 PMC4165315

[11] Sonnino S , Prinetti A . Membrane domains and the “lipid raft” concept. Curr Med Chem. 2013;20(1):4–21. 23150999

[12] Fan J , Sammalkorpi M , Haataja M . Influence of nonequilibrium lipid transport, membrane compartmentalization, and membrane proteins on the lateral organization of the plasma membrane. Phys Rev. 2010;81(1):011908. 10.1103/PhysRevE.81.011908 20365400

[13] Trajkovic K , Hsu C , Chiantia S , et al. Ceramide triggers budding of exosome vesicles into multivesicular endosomes. Science. 2008;319(5867):1244–1247. 18309083 10.1126/science.1153124

[14] Mohamed A , Robinson H , Erramouspe PJ , Hill MM . Advances and challenges in understanding the role of the lipid raft proteome in human health. Expert Rev Proteomics. 2018;15(12):1053–1063. 30403891 10.1080/14789450.2018.1544895

[15] Kosaka N , Iguchi H , Yoshioka Y , Takeshita F , Matsuki Y , Ochiya T . Secretory mechanisms and intercellular transfer of MicroRNAs in living cells. J Biol Chem. 2010;285(23):17442–17452. 20353945 10.1074/jbc.M110.107821 PMC2878508

[16] Škalamera D , Dahmer M , Purdon AS , et al. Generation of a genome scale lentiviral vector library for EF1α promoter‐driven expression of human ORFs and identification of human genes affecting viral titer. PLoS One. 2012;7(12):e51733. 23251614 10.1371/journal.pone.0051733 PMC3520899

[17] Risso D , Ngai J , Speed TP , Dudoit S . Normalization of RNA‐seq data using factor analysis of control genes or samples. Nat Biotechnol. 2014;32:896. 25150836 10.1038/nbt.2931 PMC4404308

[18] Love MI , Huber W , Anders S . Moderated estimation of fold change and dispersion for RNA‐seq data with DESeq2. Genome Biol. 2014;15(12):550. 25516281 10.1186/s13059-014-0550-8 PMC4302049

[19] Hughes JD , Estep PW , Tavazoie S , Church GM . Computational identification of cis‐regulatory elements associated with groups of functionally related genes in Saccharomyces cerevisiae. J Mol Biol. 2000;296(5):1205–1214. 10698627 10.1006/jmbi.2000.3519

[20] Harbison CT , Gordon DB , Lee TI , et al. Transcriptional regulatory code of a eukaryotic genome. Nature. 2004;431:99. 15343339 10.1038/nature02800 PMC3006441

[21] Cook KB , Kazan H , Zuberi K , Morris Q , Hughes TR . RBPDB: a database of RNA‐binding specificities. Nucleic Acids Res. 2011;39(Database issue):D301–D308. 21036867 10.1093/nar/gkq1069 PMC3013675

[22] Fabregat A , Jupe S , Matthews L , et al. The reactome pathway knowledgebase. Nucleic Acids Res. 2018;46(D1):D649–D655. 29145629 10.1093/nar/gkx1132 PMC5753187

[23] Thomas PD . PANTHER: a library of protein families and subfamilies indexed by function. Genome Res. 2003;13(9):2129–2141. 12952881 10.1101/gr.772403 PMC403709

[24] Knott GJ . *Structural Insights into DBHS Protein Dimerisation and Nucleic Acid Binding*. Perth: School of Chemistry and Biochemistry. The Univeristy of Western Australia; 2016.

[25] Robinson HR , Hill MMC , Cristino AS . Subcellular localization of MicroRNAs by MicroRNA in situ hybridization (miR‐ISH). In: Batra J , Srinivasan S , editors. Theranostics: Methods and Protocols. New York: Springer; 2019:159–169. 10.1007/978-1-4939-9769-5_11 31482455

[26] Bailey TL , Boden M , Buske FA , et al. MEME suite: tools for motif discovery and searching. Nucleic Acids Res. 2009;37(suppl_2):W202–W208. 19458158 10.1093/nar/gkp335 PMC2703892

[27] Santangelo L , Giurato G , Cicchini C , et al. The RNA‐binding protein SYNCRIP is a component of the hepatocyte exosomal machinery controlling MicroRNA sorting. Cell Rep. 2016;17(3):799–808. 27732855 10.1016/j.celrep.2016.09.031

[28] Villarroya‐Beltri C , Gutiérrez‐Vázquez C , Sánchez‐Cabo F , et al. Sumoylated hnRNPA2B1 controls the sorting of miRNAs into exosomes through binding to specific motifs. Nat Commun. 2013;4:2980. 24356509 10.1038/ncomms3980 PMC3905700

[29] Otoshi T , Tanaka T , Morimoto K , Nakatani T . Cytoplasmic accumulation of heterogeneous nuclear ribonucleoprotein k strongly promotes tumor invasion in renal cell carcinoma cells. PLoS One. 2016;10(12):e0145769. 10.1371/journal.pone.0145769 PMC4699215 26713736

[30] Barboro P , Salvi S , Rubagotti A , et al. Prostate cancer: prognostic significance of the association of heterogeneous nuclear ribonucleoprotein K and androgen receptor expression. Int J Oncol. 2014;44(5):1589–1598. 24626777 10.3892/ijo.2014.2345

[31] Wang LG , Johnson EM , Kinoshita Y , et al. Androgen receptor overexpression in prostate cancer linked to Purα loss from a novel repressor complex. Cancer Res. 2008;68(8):2678. 18413735 10.1158/0008-5472.CAN-07-6017

[32] Barboro P , Repaci E , Rubagotti A , et al. Heterogeneous nuclear ribonucleoprotein K: altered pattern of expression associated with diagnosis and prognosis of prostate cancer. Br J Cancer. 2009;100(10):1608–1616. 19401687 10.1038/sj.bjc.6605057 PMC2696760

[33] Mukhopadhyay NK , Kim J , Cinar B , et al. Heterogeneous nuclear ribonucleoprotein K is a novel regulator of androgen receptor translation. Cancer Res. 2009;69(6):2210. 19258514 10.1158/0008-5472.CAN-08-2308 PMC2659763

[34] Maurizi A , Rucci N . The osteoclast in bone metastasis: player and target. Cancers (Basel). 2018;10(7):218. 10.3390/cancers10070218 PMC6071064 29954079

[35] Huang X , Xiong X , Liu J , Zhao Z , Cen X . MicroRNAs‐containing extracellular vesicles in bone remodeling: an emerging frontier. Life Sci. 2020;254:117809. 32428598 10.1016/j.lfs.2020.117809

[36] Fan X , Xiong H , Wei J , et al. Cytoplasmic hnRNPK interacts with GSK3beta and is essential for the osteoclast differentiation. Sci Rep. 2015;5:17732. 26638989 10.1038/srep17732 PMC4671015

[37] Choi HS , Hwang CK , Song KY , Law P‐Y , Wei L‐N , Loh HH . Poly(C)‐binding proteins as transcriptional regulators of gene expression. Biochem Biophys Res Commun. 2009;380(3):431–436. 19284986 10.1016/j.bbrc.2009.01.136 PMC2657093

[38] Lee H , Li C , Zhang Y , Zhang D , Otterbein LE , Jin Y . Caveolin‐1 selectively regulates microRNA sorting into microvesicles after noxious stimuli. J Exp Med. 2019;216(9):2202–2220. 31235510 10.1084/jem.20182313 PMC6719430

[39] Ariotti N , Wu Y , Okano S , et al. An inverted CAV1 (caveolin 1) topology defines novel autophagy‐dependent exosome secretion from prostate cancer cells. Autophagy. 2020. 10.1080/15548627.2020.1820787. PMC8496722 32897127

[40] Shaikh SR , Kinnun JJ , Leng X , Williams JA , Wassall SR . How polyunsaturated fatty acids modify molecular organization in membranes: insight from NMR studies of model systems. Biochim Biophys Acta. 2015;1848(1, Part B):211–219. 24820775 10.1016/j.bbamem.2014.04.020

[41] Henkhaus RS , Roy UKB , Cavallo‐Medved D , Sloane BF , Gerner EW , Ignatenko NA . Caveolin‐1‐mediated expression and secretion of Kallikrein 6 in colon cancer cells. Neoplasia. 2008;10(2):140–148. 18283336 10.1593/neo.07817 PMC2244689

[42] Cavallo‐Medved D . Caveolin‐1 mediates the expression and localization of cathepsin B, pro‐urokinase plasminogen activator and their cell‐surface receptors in human colorectal carcinoma cells. J Cell Sci. 2005;118(7):1493. 15769846 10.1242/jcs.02278

[43] Selga E , Morales C , Noé V , Peinado MA , Ciudad CJ . Role of Caveolin 1, E‐Cadherin, Enolase 2 and PKCalpha on resistance to methotrexate in human HT29 colon cancer cells. BMC Med Genet. 2008;1(1):35. 10.1186/1755-8794-1-35 PMC2527490 18694510

[44] Arielly SS , Ariel M , Yehuda R , Scigelova M , Yehezkel G , Khalaila I . Quantitative analysis of caveolin‐rich lipid raft proteins from primary and metastatic colorectal cancer clones. J Proteomics. 2012;75(9):2629–2637. 22484058 10.1016/j.jprot.2012.03.011

[45] Beckler MD , Higginbotham JN , Franklin JL , et al. Proteomic analysis of exosomes from mutant KRAS colon cancer cells identifies intercellular transfer of mutant KRAS. Mol Cell Biochem. 2013;12(2):343–355. 10.1074/mcp.M112.022806 PMC3567858 23161513

[46] Hurwitz SN , Rider MA , Bundy JL , Liu X , Singh RK , Meckes DG . Proteomic profiling of NCI‐60 extracellular vesicles uncovers common protein cargo and cancer type‐specific biomarkers. Oncotarget. 2016;7(52):86999–87015. 27894104 10.18632/oncotarget.13569 PMC5341331

[47] Whiteside TL . Exosomes and tumor‐mediated immune suppression. J Clin Invest. 2016;126(4):1216–1223. 26927673 10.1172/JCI81136 PMC4811135

[48] Bhome R , Del Vecchio F , Lee G‐H , et al. Exosomal microRNAs (exomiRs): small molecules with a big role in cancer. Cancer Lett. 2018;420:228–235. 29425686 10.1016/j.canlet.2018.02.002 PMC5831981

[49] Hayer A , Stoeber M , Ritz D , Engel S , Meyer HH , Helenius A . Caveolin‐1 is ubiquitinated and targeted to intralumenal vesicles in endolysosomes for degradation. J Cell Biol. 2010;191(3):615–629. 21041450 10.1083/jcb.201003086 PMC3003328

[50] Simón L , Campos A , Leyton L , Quest AFG . Caveolin‐1 function at the plasma membrane and in intracellular compartments in cancer. Cancer Metastasis Rev. 2020;39(2):435–453. 32458269 10.1007/s10555-020-09890-x PMC7311495

[51] Shi Y , Tan S‐H , Ng S , et al. Critical role of CAV1/caveolin‐1 in cell stress responses in human breast cancer cells via modulation of lysosomal function and autophagy. Autophagy. 2015;11(5):769–784. 25945613 10.1080/15548627.2015.1034411 PMC4509445

[52] Zhou Y , Hancock JF . Ras nanoclusters: versatile lipid‐based signaling platforms. Biochim Biophys Acta. 2015;1853(4):841‐849. 25234412 10.1016/j.bbamcr.2014.09.008

[53] Termini CM , Gillette JM . Tetraspanins function as regulators of cellular signaling. Front Cell Dev Biol. 2017;5:34. 28428953 10.3389/fcell.2017.00034 PMC5382171

[54] Molendijk J , Robinson H , Djuric Z , Hill MM . Lipid mechanisms in hallmarks of cancer. Mol Omics. 2020;16(1):6‐18. 31755509 10.1039/c9mo00128j PMC7184895

[55] Moon H , Ruelcke JE , Choi E , et al. Diet‐induced hypercholesterolemia promotes androgen‐independent prostate cancer metastasis via IQGAP1 and caveolin‐1. Oncotarget. 2015;6(10):7438–7453. 25924234 10.18632/oncotarget.3476 PMC4480691

[56] Ha M , Kim VN . Regulation of microRNA biogenesis. Nat Rev Mol Cell Biol. 2014;15:509. 25027649 10.1038/nrm3838

[57] Leung AKL . The whereabouts of microRNA actions: cytoplasm and beyond. Trends Cell Biol. 2015;25(10):601–610. 26410406 10.1016/j.tcb.2015.07.005 PMC4610250

[58] Bottini S , Hamouda‐Tekaya N , Mategot R , et al. Post‐transcriptional gene silencing mediated by microRNAs is controlled by nucleoplasmic Sfpq. Nat Commun. 2017;8(1):1189. 29084942 10.1038/s41467-017-01126-x PMC5662751

[59] Treiber T , Treiber N , Plessmann U , et al. A compendium of RNA‐binding proteins that regulate MicroRNA biogenesis. Mol Cell. 2017;66(2):270–284.e13. 28431233 10.1016/j.molcel.2017.03.014

[60] Moujalled D , James JL , Yang S , et al. Phosphorylation of hnRNP K by cyclin‐dependent kinase 2 controls cytosolic accumulation of TDP‐43. Hum Mol Genet. 2015;24(6):1655–1669. 25410660 10.1093/hmg/ddu578

[61] Habelhah H , Shah K , Huang L , et al. ERK phosphorylation drives cytoplasmic accumulation of hnRNP‐K and inhibition of mRNA translation. Nat Cell Biol. 2001;3(3):325–330. 11231586 10.1038/35060131

[62] Hope NR , Murray GI . The expression profile of RNA‐binding proteins in primary and metastatic colorectal cancer: relationship of heterogeneous nuclear ribonucleoproteins with prognosis. Hum Pathol. 2011;42(3):393–402. 21194727 10.1016/j.humpath.2010.08.006

[63] Watahiki A , Wang Y , Morris J , et al. MicroRNAs associated with metastatic prostate cancer. PLoS One. 2011;6(9):e24950. 21980368 10.1371/journal.pone.0024950 PMC3184096

[64] Liu Y , Gervasi C , Szaro BG . A crucial role for hnRNP K in axon development in Xenopus laevis. Development. 2008;135(18):3125–3135. 18725517 10.1242/dev.022236

[65] Vidaki M , Drees F , Saxena T , et al. A requirement for mena, an actin regulator, in local mRNA translation in developing neurons. Neuron. 2017;95(3):608–622.e5. 28735747 10.1016/j.neuron.2017.06.048 PMC5616167

[66] Proepper C , Steinestel K , Schmeisser MJ , et al. Heterogeneous nuclear ribonucleoprotein K interacts with Abi‐1 at postsynaptic sites and modulates dendritic spine morphology. PLoS One. 2011;6(11):e27045. 22102872 10.1371/journal.pone.0027045 PMC3216941

