# Supplementary material for: Caveolin‐1‐driven membrane remodelling regulates hnRNPK‐mediated exosomal microRNA sorting in cancer
Source: Clin Transl Med. 2021 Apr 8;11(4):e381. doi: 10.1002/ctm2.381 (PMC8031663; doi:10.1002/ctm2.381)
Supplement: Supplementary file 1 — Supporting materials and methods: SWATH library generation and sample processing. Figure S1: hnRNPK translocates between MVB in PC3‐CONT cells to mitochondrial and ER regions in PC3‐CAVIN1 cells, related to Figure 2. CD9 (MVB marker), ERp44 (ER marker) and VDAC4 (mitochondrial marker) localisation (green) relative to hnRNPK (red) in PC3‐CONT (A) and PC3‐CAVIN1 (B) cells. DAPI (blue) displays nuclear localisation. Images taken from two to four biological replicates. Scale bars are 10 μm. Boxed areas in top panel indicate ROI for co‐localisation analysis. Data retrieved from analysis are available in Table S3. Figure S2: hnRNPK only translocates to MVB compartments in CAV1 expressing LNCaP cells, related to Figure 2. Images show hnRNPK (red) localisation in LNCaP‐CONT (A), LNCaP‐CAV1 (B) and LNCaP‐CAVIN1 (C) cell lines, relative to CD9 (green) and DAPI (blue) staining. Images were taken from three biological replicates. Scale bar denotes 10 μm. Boxed regions in composite (merge) indicate ROIs for co‐localisation analysis. Data retrieved from analysis are available in Table S3. Figure S3: hnRNPK only translocates to MVB compartments in CAV1 expressing HEK293 cells, related to Figure 2. Additional images showing hnRNPK (red) and CD9 (green) localisation in HEK293‐CONT (A) and HEK293‐CAV1 (B) cell lines, and DAPI (blue) staining. Images were taken from three biological replicates. Scale bar denotes 10 μm. Boxed regions in composite (merge) indicate ROIs for co‐localisation analysis. Data retrieved from analysis are available in Table S3. Figure S4: Motif‐containing mature miR‐30a co‐localised with hnRNPK in PC3‐CONT cells, related to Figure 5. Mature motif‐containing miR‐30a‐5p, precursor miR‐30a (miR‐30a‐loop), a motif‐absent miR‐363‐3p and a scrambled miRNA (green) control were visualised using miR‐ISH and co‐localised with hnRNPK (red) in PC3‐CONT and PC3‐CAVIN1 cells. Ten ROIs were used for co‐localisation analysis (right), where applicable. Data retrieved from analy [file CTM2-11-e381-s003.pdf]

Supporting information

Caveolin-1-driven membrane remodelling regulates hnRNPK-mediated exosomal microRNA sorting in cancer

Running title: CAV1 drives hnRNPK loading of exosomal miRNA

Harley Robinson[1,2], Jayde E Ruelcke[1], Amanda Lewis[3], Charles S. Bond[3], Archa H. Fox[3,4,5], Vandhana Bharti[2], Shivangi Wani[2], Nicole Cloonan[2] , Andrew Lai[6], David Margolin[7], Li Li[7], Carlos Salomon[6,7,8], Renee S. Richards[2], Aine Farrell[6], Robert A Gardiner[6], Robert G. Parton[9,11], Alexandre S. Cristino [1,10*], Michelle M Hill [1,2*]

Supporting figures in this document:

Supporting materials and methods: SWATH library generation and sample processing.

Figure S1: hnRNPK translocates between MVB in PC3-CONT cells to mitochondrial and ER regions in PC3-CAVIN1 cells, related to figure 2.

Figure S2: hnRNPK only translocates to MVB compartments in CAV1 expressing LNCaP cells, related to figure 2.

Figure S3: hnRNPK only translocates to MVB compartments in CAV1 expressing HEK293 cells, related to figure 2.

Figure S4: Motif-containing mature miR-30a co-localised with hnRNPK in PC3-CONT cells, related to figure 5.

Figure S5: Membrane raft alterations modify hnRNPK localisation in PC3 cells, related to figure 6.

Supporting tables in separate documents:

Table S1: Patient information tables, related to Methods.

Table S2: DESeq2 results for cellular miRNA (Tab 1), EVs (Tab 2), and the associated classification analysis (Tab 3), Motif Enrichment (Tab 4) and Motif scanning (Tab 5) related to figure 1.

Table S3: Co-localisation analysis between hnRNPK and CD9 (Tab 1), and hnRNPK and miRNA targets (Tab 2), related to figures 2 and 5.

Table S4: Quantitative analyses for raft disruption treatments for DRM protein abundance (Tab 1) and hnRNPK level in cytoplasmic and nuclear fractions (Tab 2), related to figure 6.

Table S5: SWATH data from serum EVs extracted from early and metastatic CRC patients, relating to Figure 7.

Table S6: Complete list of oligonucleotides used in this paper.

Supporting Methods and Materials.

SWATH spectral library generation

EV samples were lysed in 4% SDS, 50 mM Tris, pH 8.0 with 10 min sonication in a bath sonicator (Elmasonic). Pooled EV lysate from colorectal cancer plasma (30 µg) was separated on a 4-12% acrylamide/bisacrylamide gel (Thermo Fisher) then the gel was stained with Simply Blue for 1 hr. Ten gel bands were excised from each sample lane and were destained in 50% acetonitrile (v/v)/ 50 mM ammonium bicarbonate (ABC). The cysteine bonds were reduced with 10 mM dithiothreitol (DTT, Sigma) in 100 mM ABC at 56°C for 30 min and alkylated with 50 mM iodoacetamide (IAA, Sigma) for 20 min in the dark. Trypsin solution at 20 µg/mL (Promega) in 50 mM ammonium bicarbonate were added to just cover the gel pieces before an overnight incubation at 37°C The resulting tryptic peptides were desalted using the SOLAµ HRP 96 well plates (Thermo Fisher).

Tryptic peptides (2 µg) were analysed on an AB SCIEX Triple TOF spectrometer (ABSCIEX, Redwood City, CA) coupled to a Nano Ultra 1D+ HPLC system (Eksigent, Redwood City, CA) with reversed phase CHROMXP C18CL 3 µm, 120^{0}A, 150 x 0.075 mm (Eksigent, Redwood City, CA) analytical column and trap column (CHROMXP C18CL 5 µm, 10 x 0.3 mm; Eksigent, Redwood City, CA). On-column wash was performed for 15 min at 3µL/min, while the analytical flow rate was 250 nL/min. LC gradient started with 95% mobile phase A (H_2O/0.1% FA), 5% B (ACN/0.1%FA) at 0 min and increase to 10%B over for 2 min and then a 58-min linear gradient to 40% B followed by 50% B for 5 min. Mobile phase B was then increased from 50% to 95% over 10 min followed by column wash at 95% B for 15 min and re-equilibrated with 5% Buffer B for 6 min.

The mass spectrometer was operated in data-dependent acquisition (DDA) mode with the following parameters: ion spray voltage (IAVF) of 2200V, curtain gas of (CUR) 25, ion source gas of (GAS1) 10 and de-clustering potential (DP) of 70. Full scan (survey scan) mass spectra were acquired for 200 ms in high sensitivity mode from 300 to 1800 m/z followed by collision-induced dissociation (CID) data-dependent product ion scan (MS/MS) of most abundant ions (Top 18) from survey scan. Accumulation time was set to 100 ms for product ion scan (MS/MS) with a collision energy (CE) of 10. Target ions already selected for the MS/MS were dynamically excluded for 15 s after two occurrences. The minimum threshold was set 50 counts per seconds (cps) for ion selection and ion with charge state 2 to 4 were selected for MS/MS.

SWATH sample processing

Protein concentration of individual EV samples were estimated using Bradford assay (BioRad) then 20 µg was digested using filter-aided sample preparation (FASP) method. The lysates were diluted in 5 volumes of 8 M urea/ 50 mM Tris pH 8.5 and placed in a 30 kDa Centrifugal Filter Units (Pall). Proteins were reduced with 100 mM DTT for 1 hr at room temperature then alkylated with 50 mM IAA for 20 min in the dark. Reagents were removed by centrifugation at 10,000 x g at 20°C for 5 min. Trypsin in ABC was added at a 1:50 enzyme:protein ratio prior to an overnight incubation at 37°C. Peptides were then collected by adding 50 µL ABC to the filter before centrifugation at 10,000 x g at 20°C for 15 min. The resulting tryptic peptides were desalted using the SOLAµ HRP 96 well plates (Thermo Fisher).

Sequential Windowed Acquisition of All Theoretical Fragment Ion Mass Spectra (SWATH™) data acquisition used the same LC and ion source settings as described above, with a total of 32 isolation windows from 400 -1200 Da with a fixed window of 25 Da.

The data-dependent acquisition (DDA)-MS raw file was subjected to database searches using ProteinPilot software version 4.5b (Sciex, Framingham, MA). Raw data files from all fractions were analysed using the ProteinPilot software. For each sample, the raw data was searched against a

human SwissProt database from UNIPROT database (http://www.uniprot.org/proteomes/UP000000589). A global false discovery rate (FDR) of 1% was used as the threshold for the number of proteins for import.

For SWATH processing, the SWATH Acquisition Microapp (version 2.0) within PeakView (RRID: SCR_015786; version 2.2) was used. Within the Microapp, a setting of three peptides per protein, four transitions per peptide, peptide confidence threshold corresponding to 1% global FDR. The retention time was then manually realigned with a minimum of five peptides that had consistently high signal intensities and distributed along the time axis. The resulting peak area for each protein after SWATH processing was exported to MarkerView (version 1.3.1; Sciex) with the resulting data normalized using the most likely ratio method. The proteomics data have been deposited in the PRIDE PRoteomics IDEntifications (PRIDE) database under the accession number PXD013740.

From the SWATH processing of the patient EV population, 4 biological replicates were obtained for each grade of colorectal cancer. Grade I and II were grouped and termed as early CRC, and grades III and IV were collectively termed metastatic CRC, making 8 biological replicates used in the early versus metastatic CRC analysis (n=8). Fold changes were generated using the mean normalised intensity for each protein comparing between early and metastatic CRC, and $\log_2$ transformed. For graphical purposes, p-values were converted to -$\log_2$p-values and plotted against $\log_2$FC using R studio to generate volcano plots. Statistically significant proteins (p-value < 0.05) were coloured red and those proteins increased in metastatic CRC (positive log2FC value) were labelled with gene name for visibility. Using GraphPad Prism, early and metastatic CRC normalized intensities for hnRNPK were graphed using box and whisker plots which displays range of observation, median and upper and lower quartiles.

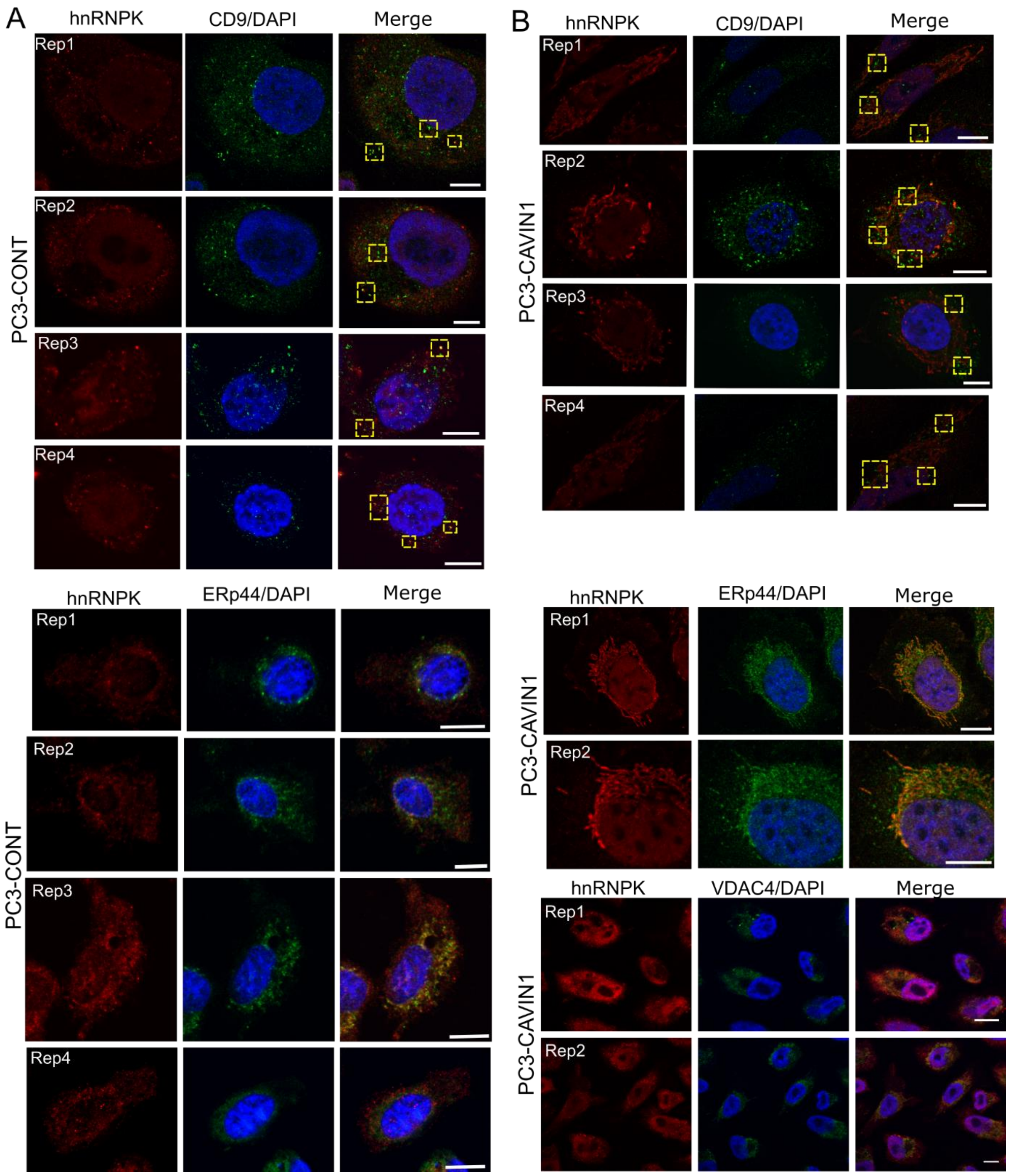

Figure S1: hnRNPK translocates between MVB in PC3-CONT cells to mitochondrial and ER regions in PC3-CAVIN1 cells, related to figure 4. CD9 (MVB marker), ERp44 (ER marker) and VDAC4 (mitochondrial marker) localisation (green) relative to hnRNPK (red) in PC3-CONT **(A)** and PC3-CAVIN1 **(B)** cells. DAPI (blue) displays nuclear localisation. Images taken from 2-4 biological replicates. Scale bars are 10 µm. Boxed areas in top panel indicate ROI for co-localisation analysis. Data retrieved from analysis available in Table S3.

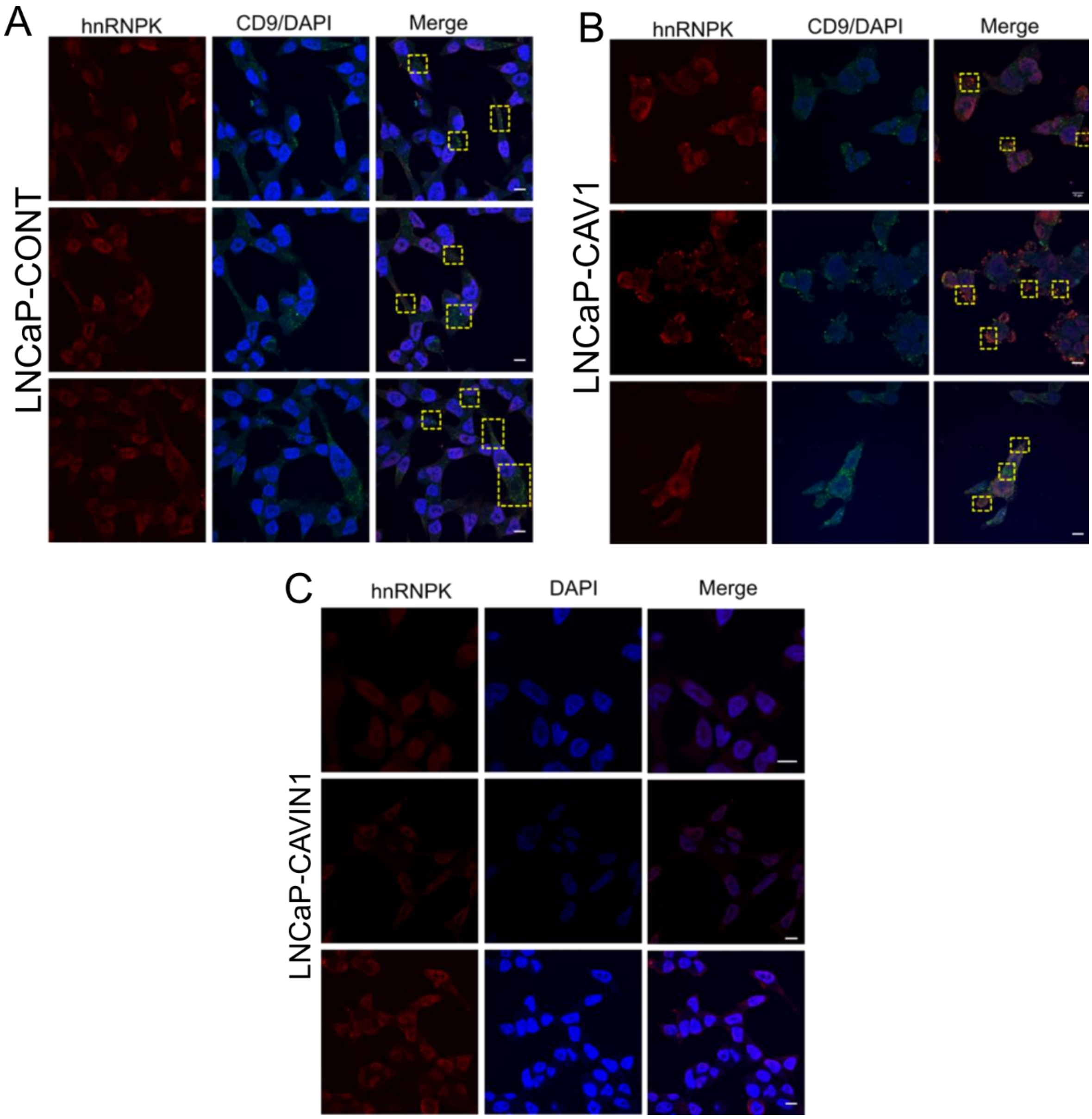

Figure S2: hnRNPK only translocates to MVB compartments in CAV1 expressing LNCaP cells, related to figure 4. Images show hnRNPK (red) localisation in LNCaP-CONT **(A),** LNCaP-CAV1 **(B)** and LNCaP-CAVIN1 **(C)** cell lines, relative to CD9 (green) and DAPI (blue) staining. Images were taken from 3 biological replicates. Scale bar denotes 10 µm. Boxed regions in composite (merge) indicate ROIs for co-localisation analysis. Data retrieved from analysis available in Table S3.

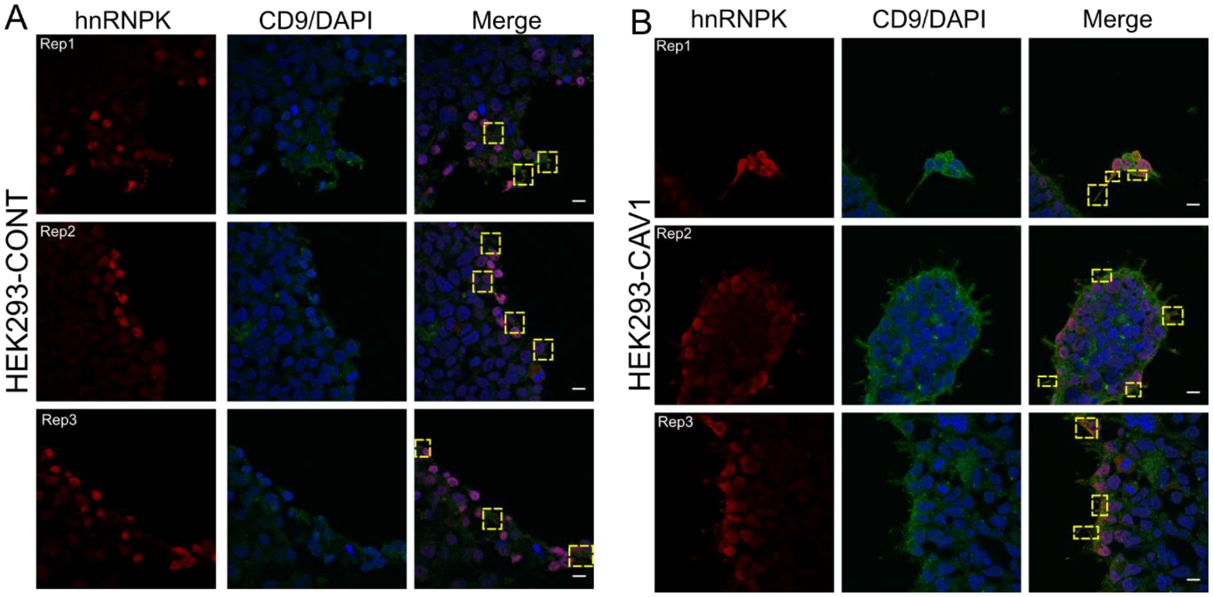

Figure S3: hnRNPK only translocates to MVB compartments in CAV1 expressing HEK293 cells, related to figure 4. Additional images showing hnRNPK (red) and CD9 (green) localisation in HEK293-CONT **(A)** and HEK293-CAV1 **(B)** cell lines, and DAPI (blue) staining. Images were taken from 3 biological replicates. Scale bar denotes 10 µm. Boxed regions in composite (merge) indicate ROIs for co-localisation analysis. Data retrieved from analysis available in Table S3.

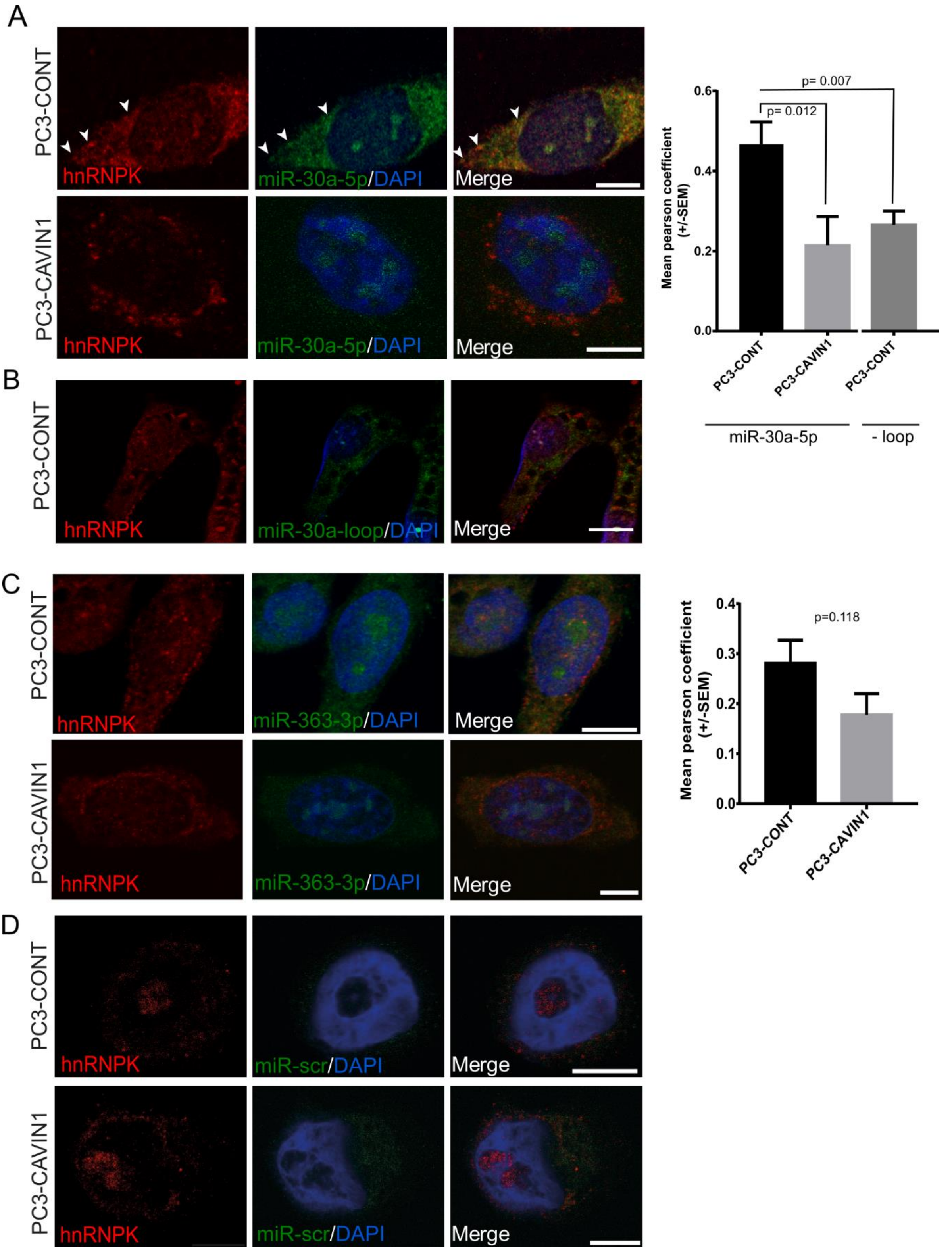

Figure S4: Motif-containing mature miR-30a co-localised with hnRNPK in PC3-CONT cells, related to figure 3. Mature motif-containing miR-30a-5p, precursor miR-30a (miR-30a-loop), a motif-absent miR-363-3p and a scrambled miRNA (green) control were visualised using miR-ISH and co-localised with hnRNPK (red) in PC3-CONT and PC3-CAVIN1 cells. 10 ROIs were used for co-localisation analysis (right), where applicable.

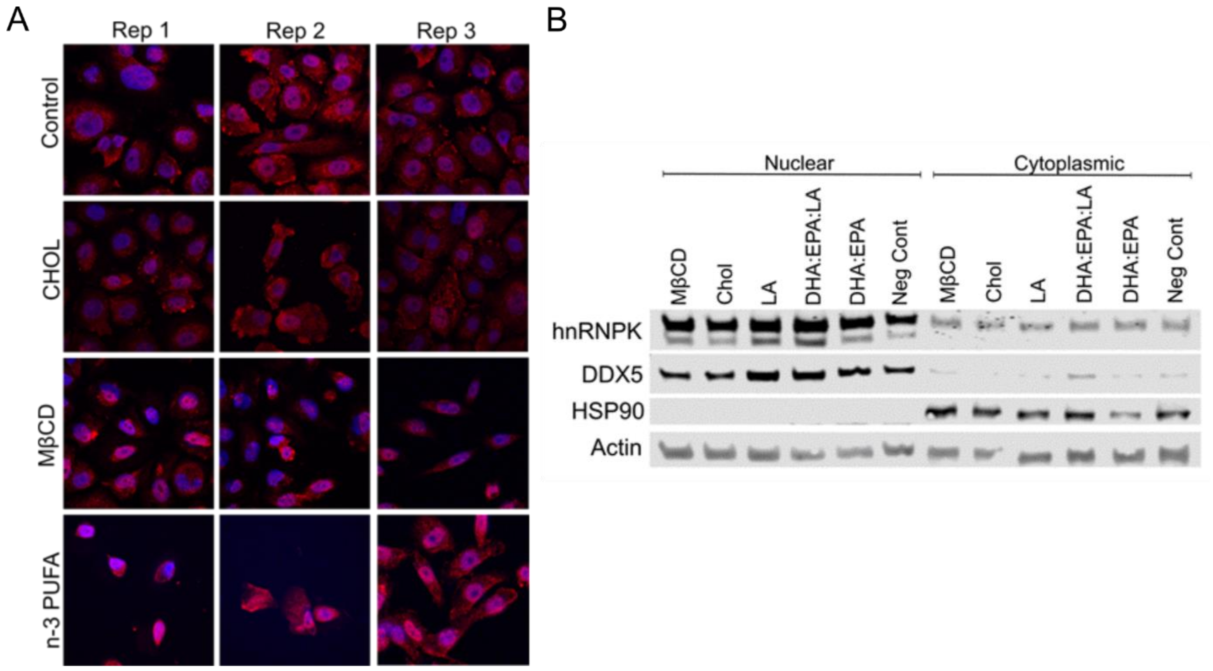

Figure S5: Membrane raft alterations modify hnRNPK localisation in PC3 cells, related to figure 5. **A)** hnRNPK (red) and DAPI (blue) were visualised after exposure of PC3 cells to 500µM MβCD, 50µM cholesterol (CHOL), or n-3 PUFA (30µM DHA: 45µM EPA) in LPDS/RPMI1640 for 24hrs across 3 biological replicates. **B)** Western immunoblot comparing nuclear and cytoplasmic signal for hnRNPK, DDX5 (nuclear marker), HSP90 (cytoplasmic marker) and actin (loading control). 10µg protein loaded into each well. Quantitation of each band used for analysis is available in Table S4 for all replicates.
